# Scoping Review and Bibliometric Analysis of the Most Influential Publications in Achalasia Research from 1995 to 2020

**DOI:** 10.1155/2021/8836395

**Published:** 2021-02-04

**Authors:** Huifang Xia, Shali Tan, Shu Huang, Peiling Gan, Chunyu Zhong, Muhan Lü, Yan Peng, Xian Zhou, Xiaowei Tang

**Affiliations:** ^1^Department of Gastroenterology, Affiliated Hospital of Southwest Medical University, Luzhou, China; ^2^Department of Gastroenterology, The People's Hospital of Lianshui, Huaian, China

## Abstract

**Objective:**

To identify and evaluate characteristics of the most influential articles in achalasia research during the period 1995-2020.

**Methods:**

Articles in Scopus, Web of Science Core Collection (WoSCC), and PubMed were scanned from 1995 to 2020 with achalasia as the keyword. We retrieved the articles that met all criteria by descending order after using EndNote to remove the duplicated references. Our bibliometric analysis highlighted publication year, country, journals, and networks of keywords.

**Results:**

Fifteen percent of the top 100 most-cited articles were published in *Annals of Surgery.* They were performed in 15 countries, and most (*n* = 55) were from the USA. The number of citations of the 482 articles ranged from 30 to 953, 38 of which had been published in *American Journal of Gastroenterology*. Those articles were from 31 countries, and most of the studies (*n* = 217) had been performed in the USA. Most of articles (*n* = 335) were clinical research. Treatments were hotspots in the field of achalasia in the past years. The most influential title words were “achalasia,” “esophagomyotomy,” “pneumatic dilation,” and “lower esophageal sphincter.”

**Conclusion:**

Our study offers a historical perspective on the progress of achalasia research and identified the most significant evolution in this field. Results showed treatment was the most influence aspect in achalasia.

## 1. Introduction

There is no standard or established method of quickly and effectively accessing influential publications of medical research [1]. As the number of articles which researchers need to be familiar with increases so does the importance of selective searching and generalization. Even though medical knowledge is continually advancing and new articles are published daily, the impact of articles published in the past is not decreased. Accurately determining the influence of medical papers is vital for guiding decisions in clinical practice and improving physicians' ability to quickly find important articles in a specific scientific research field. Articles of relevance to other studies are often cited in subsequent papers, so the most widely used and useful method for measuring the impact of research activity is identifying the number of citations in the published literature.

Achalasia is a rare esophageal motility disorder. The condition was first described in 1672 by Sir Thomas Willis, and Hurt and Rake coined the term achalasia, which means “failure to relax” in 1929 [2]. The symptoms include dysphagia to solids and liquids, substernal chest pain during meals, heartburn, aspiration pneumonia, regurgitation of food or saliva, and/or weight loss [3]. The diagnosis, prevention, and treatment of achalasia are well investigated, but the pathogenesis has not yet been completely described. Efforts to fully understand this disease are ongoing, and this study is aimed at identifying and evaluating the landmark articles in achalasia research.

## 2. Methods

### 2.1. Scoping Review

Scoping review is a form of knowledge synthesis, which combines and integrates a variety of study designs to summarize and synthesize evidence comprehensively. The aim is to provide information for practices, plans, and policies and to provide directions for future research priorities [4]. This study was abided by the Preferred Reporting Items for Systematic reviews and Meta-Analyses extension for Scoping Reviews (PRISMA-ScR) Checklist [5]. The Arksey and O'Malley approach framework was adopted, which summarized five stages for reporting a scoping review.

#### 2.1.1. Identifying the Research Question

What is known from the existing literature in the field of achalasia research?

#### 2.1.2. Identifying of Databases and Relevant Studies

We followed the PRISMA-ScR [5]. Articles were retrieved from three databases (including WoSCC, Scopus, and PubMed) with the time span restricting from 1995 to 2020, using the search term “achalasia” on November 15, 2020. EndNote (X9 Edition, New York, USA) was used to remove the duplicated references. We only included articles published in English with 30 or more citations.

#### 2.1.3. Study Selection

Two reviewers selected the articles with predefined inclusion and exclusion criteria (Table 1) independently (Huifang Xia and Shali Tan). Any discrepancy was resolved by discussion. The filtering methods included the time span (1995 to 2020), and language was limited in English. And the preliminary filtering through title and abstract to exclude the irrelevant articles, the final screening was done by reading the full text.

### 2.2. Bibliometric Analysis

The selection of study was in accordance with the scoping review. The qualified articles were ranked in a descending order by the citation numbers after removing duplications. Data regarding journals, publication year, country, and author were collected.

Impact factor, CiteScore, Eigenfactor score (ES), Essential Science Indicators (ESI), and Article Influence Score (AIS) were analyzed to evaluate journals' impacts. Journals with a higher score of these indicators are generally considered to be more prestigious than those with a lower score [6]. The 100 most cited articles were analyzed by authorship considering the first and the second authors, journal name and year of publication, title, and the number of citations from the specified databases.

### 2.3. Network Analysis of Keyword Cooccurrences

Network analysis is a method used to reveal the scientific structure, the degree of subject correlation, literature retrieval, and others, starting from the topic correlation reflected in the citations. In our study, we did the network analysis of the keyword correlation in citations in the field of achalasia. The network analysis of keywords can identify a research topic by publications that are closely connected to each other in terms of citation relations [7]. By the way of network analysis, current research hotspots can be detected in the field of achalasia.

VOSviewer (version 1.6.15, Netherlands) was applied to perform network analyses. We selected “all keywords” as the unit of analysis and set minimum number of occurrences of a keyword to 5. VOSviewer is a visual tool that can generate a variety of graphs based on bibliometric relations [8]. We chose it for network analysis because of its strong graphics display ability and suitability for use with large-scale data. In the density visualization, yellow nodes indicate increased weights of the neighboring items and the size of the node increases with the number of items in the neighborhood. The color of the node changes toward blue as the weights of the neighboring items decrease.

## 3. Results

### 3.1. Scoping Review

**Figure 1:**
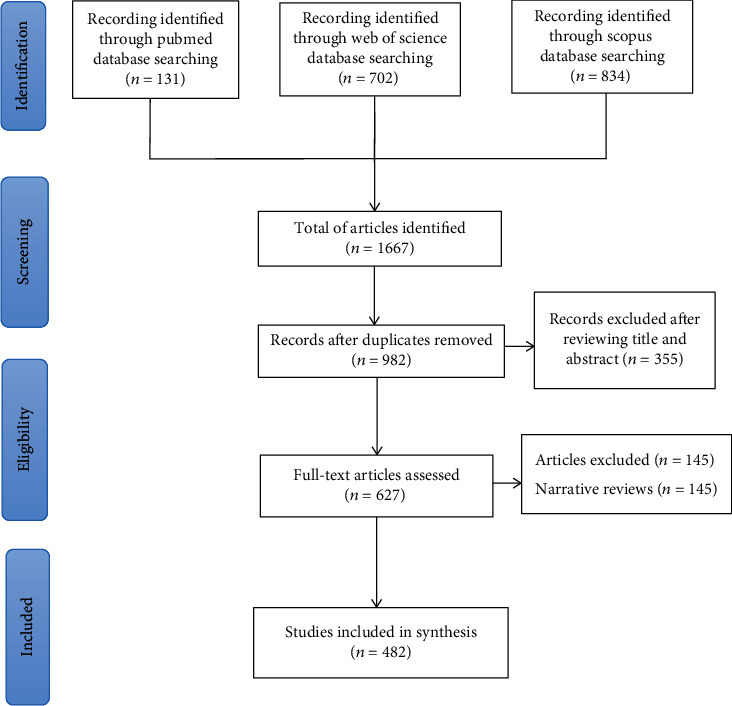
Flowchart of study.

The original search findings from the WoSCC, Scopus, and PubMed yielded 702, 834, and 131 papers, respectively. After removing the duplications, 982 articles were remained. 355 publications were excluded after screening the title and abstract. 145 studies were excluded after reviewing the full text according to the inclusion or exclusion criterion. And 482 papers meeting all identified requirements were included.  Figure 1 shows the research flowchart based on the PRISMA-ScR guidelines.

#### 3.1.1. Charting the Data

Characteristics of eligible articles are presented in Table S1, including the first authors, year of publication, number of citations, type of article, and research direction. The type of the article was classified as follows: (i) clinical research, including prospective study, retrospective study, randomized controlled trials (RCTs), and case report; (ii) review, including systematic review, literature review, and meta-analysis; (iii) guideline and consensus; and (iv) basic science research. Research directions involved several aspects: (i) etiology; (ii) diagnosis; (iii) treatment: including methods and curative effect; (iv) classification; (v) pathogenesis; (vi) other: epidemiology, demographics, pathophysiology, complication, clinical characteristics, and prognosis; and (vii) all: including great than or equal to two points mentioned above.

#### 3.1.2. Analyzing Data, Summarizing, and Reporting the Results

**Figure 2:**
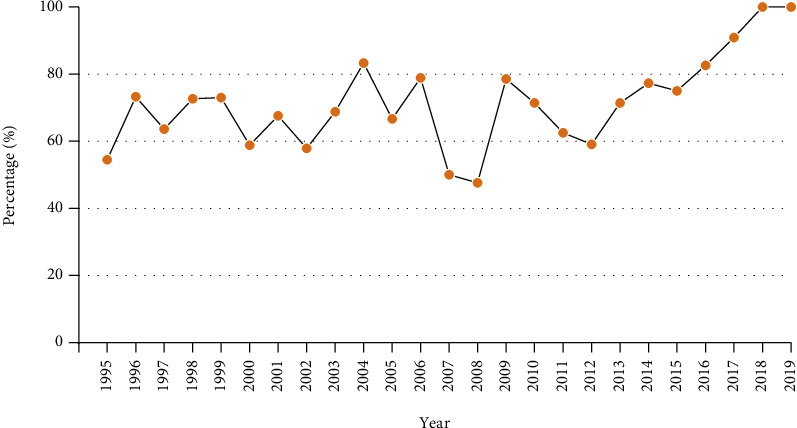
The percentage of articles about treatment in each year.

About 69.5 percent of articles were talking about the treatment of achalasia, including the methods and the curative effects; 11.4 percent of study direction were other; etiology and all accounted for approximately 6.6 percent and 6.4 percent, respectively; 3.9 percent of publications were related to diagnosis roughly; about 1.7 percent and 0.5 percent of articles were mentioned to classification and pathogenesis severally. 357 articles were clinical research, 87 articles were review, 34 publications were basic science research, and the rest of articles (*n* = 4) were guideline and consensus (Table 2).  Figure 2 shows the percentage of articles about treatment in all publications each year.

### 3.2. Bibliometric Analysis Results

**Figure 3:**
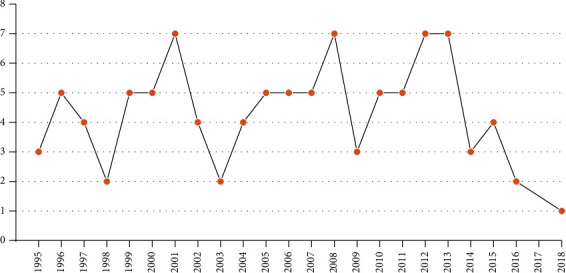
Distribution of top-100 cited articles per year.

Table 3 lists the ten most-cited articles. The mean number of citations was 563, and the range was from 332 to 953. The top-100 articles were cited 953 to 103 times. As shown in Figure 3, the top-100 articles were published between 1995 and 2018 in 29 different journals and 49% were published after the year 2006. Seventeen journals had published two or more articles. *Annals of Surgery* had the largest number of articles (15%), followed by the *Gastroenterology* (13%), *Gut* (9%), and the *American Journal of Gastroenterology* (9%). Articles published in *Annals of Surgery* had received 3001 citations. *New England Journal of Medicine* was the most cited journal, with a mean of 469 citations per article, it is also the journal with the highest scores of CiteScore, IF, ES and AIS. The *Journal of the American Medical Association* and *Gastrointestinal Endoscopy* were the journals with the least citations (Table 4). Of the top-100 cited articles, 3% were publications of basic science research, 75% were clinical research, 23% were review, and 2% were guideline or consensus report. The first author was affiliated with an academic department in most publications [9], and 22 authors had published two or more top-cited articles. The most frequently published authors are listed in Table 5, led by Pandolfino, with six articles, followed by Annese, Inoue, Kahrilas, and Zaninotto, with five each. The top-100 articles originated from 15 different countries (Table 6): 55 were from the USA, 11 from Italy, 7 from Germany, 5 from Japan, 4 each from the Netherlands and China, and two each from Switzerland and France.

**Figure 4:**
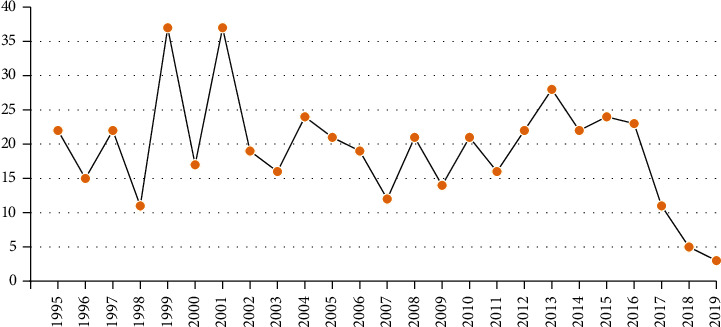
Distribution of the 482 articles per year.

Table 7 shows that the 482 articles came from 30 countries led by the USA with 217 (44.9%), followed by Italy (*n* = 51), Germany (*n* = 32), China (*n* = 30), Canada (*n* = 24), Japan (*n* = 21), and Netherlands (*n* = 12). 222 were published in 2007 or afterward (Figure 4). Twenty-eight journals had published two or more of the qualified articles: 38 were published in *American Journal of Gastroenterology*, 32 in the *Surgical Endoscopy*, 33 in the *Annals of Surgery*, 26 in *Gastrointestinal Endoscopy*, and 24 in *Digestive Diseases and Sciences* and *Annals of Surgery*, respectively. The most cited journal was *American Journal of Gastroenterology* with 3563 citations. Articles in the *New England Journal of Medicine* were the most frequently cited, with a mean of 365 per article. The overall average number of citations per article was 84 (Table 8).

### 3.3. Network Analysis of the Keyword Cooccurrences in the Eligible Articles

**Figure 5:**
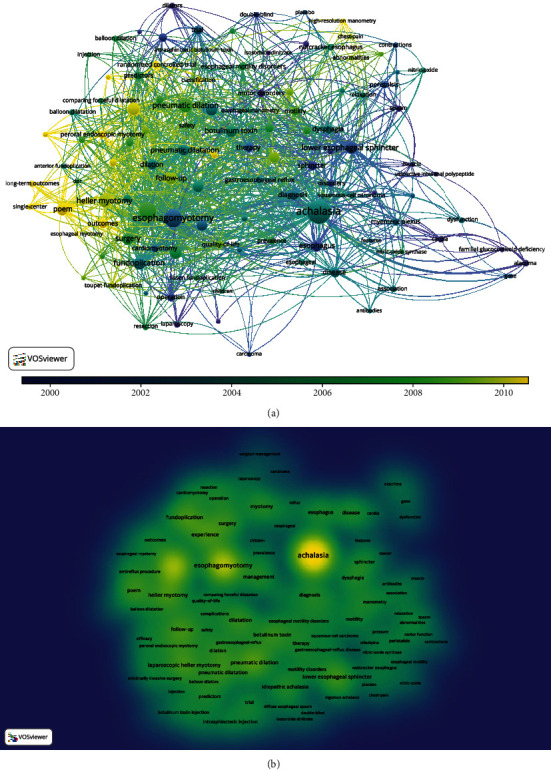
Network plot of influential keywords with five or more times in achalasia research among the 482 articles of Web of Science Core Collection: (a) overlay visualization and (b) density visualization.

The network analysis used the total word count to identify influential title words. Each word was counted once no matter how many times it appeared in the same article, and 110 words met the threshold (occurred 5 times at least) in the 1084 keywords. The overlay visualization and modularity clustering in Figure 5(a) show the score each node by its color. Links indicate keyword relevance, and the number of keyword occurrences increases the node size. Nodes are proportional in size and importance. The most influential keywords were “achalasia,” “esophagomyotomy,” “pneumatic dilation,” and “lower esophageal sphincter.” Different colors represent the time when the keyword appears. The term “esophagomyotomy” appeared in 2000. It is an early treatment for achalasia that is still used, and it occurred frequently. Peroral endoscopic myotomy (POEM) is a treatment that was developed in the early 21st century that has been rapidly adopted following a widely cited article published by Inoue in 2010. In Figure 5(b), yellow nodes indicate increased weights, and increased size indicates larger numbers, of neighboring items. Blue nodes indicate decreased weights and numbers of the neighboring items.

## 4. Discussion

The results of the scoping review and network analysis revealed that most of the articles related to achalasia were clinical research and focused on treatments, which suggested that the majority of investigators paid attention to clinical practice and the field of basic research still needed to be further explored. It also showed that treatment was a hot research topic all the time. Pneumatic dilation, peroral endoscopic myotomy, laparoscopic Heller myotomy, and endoscopic injection of botulinum toxin were the research hotspots since 2010.

Bibliometrics has been used to analyze the most frequently cited publications in clinical fields including urology, psychiatrics, ophthalmology, emergency care, orthopedics, and digestive diseases [9–14]. We believe this is the first bibliometrics study in achalasia. The top 100 articles were cited from 101 to 953 times, while the 100 most frequently cited articles on digestive diseases were cited between 853 and 4895 times [9]. Achalasia is rare condition, with a morbidity of about 1 : 100,000 cases of digestive disease and less than 5% of those occurring in children, or about 0.11 per 100,000 pediatric patients [15, 16]. That shows why there are more citations for digestive diseases as a group than for achalasia.

The classification of esophageal motility abnormalities is quite important for achalasia as it can classify the subtypes of achalasia. Four of the top ten articles focused on classification, three included the Chicago classification, and the fourth, published in 2001, divided achalasia into typical and atypical types [17]. The Chicago classification was introduced by Pandolfino et al. and is one of the most influential diagnostic criteria used in current clinical practice [18]. The Chicago classification divides achalasia into three subtypes determined by the use of high-resolution manometry [19, 20], The first version was published in 2009 following a meeting of the International high-resolution manometry (HRM) Working Group in San Diego in 2008. The second was published in 2012 and was the third most frequently cited article in this analysis [19]. The second most frequently cited article describes version 3.0 of the Chicago classification [21]. Each new version has been updated by evidence published after the older version that is relevant to the clinical interpretation of HRM studies.

Domestic and international studies have found that patients with achalasia of different subtypes have different clinical characteristics and esophageal dynamics. Type I achalasia has features of lower esophageal sphincter (LES) relaxation, absence of esophageal pressurization, and aperistalsis. Type II achalasia is the most common type and is characterized by the absence of peristalsis and intermittent periods of compartmentalized esophageal pressurization. Type III achalasia is the least common. Dysphagia is common to all three types, and a Chicago classification study published in 2018 presented evidence that in the evolution of the disease, type III is the earliest stage of achalasia, type II is an intermediate stage, and type I is the final stage [22, 23]. Subtype II is reported to have the best prognosis, followed by subtype I and subtype III, which can be difficult to treat [22, 24–26]. The Chicago classification plays an important role in predicting the prognosis and guiding the treatment of different achalasia subtypes.

The most frequently cited article was published by Inoue H in 2010; it described the use of POEM, which is a novel, revolutionary endoscopic technology for the treatment of achalasia [27]. POEM has a significant improvement of dysphagia, an improved peri- and postoperative experience, and faster postoperative recovery compared with the outcomes with older methods. Because of its advantages, the use of POEM has been adopted worldwide to treat achalasia. The first description of POEM to treat achalasia in the USA was by published by Swanstrom in 2012, who reported continuing remission of dysphagia over 11 months of follow-up in a series of 18 patients [28]. POEM may be more effective than other treatment modalities for patients with type III achalasia because it achieves a more durable myotomy than laparoscopic Heller myotomy (LHM) [29, 30].

Pneumatic dilation (PD) is an outpatient endoscopic procedure for treating achalasia and acts by disrupting the circular muscle fibers of the LES to eliminate functional obstruction at the level of the gastroesophageal junction. Seventeen of the top 100 articles (ranks 6, 7, 22, 26, 34, 35, 46, 50, 54, 57, 62, 64, 80, 81, 82, 88, and 91) described the use of PD to treat achalasia. The measurement of intracavitary pressure measurement to evaluate the effectiveness of PD treatment was first described in 1971, and other frequently cited articles described the use of esophageal sphincter pressure to guide the next steps of treatment as well as the use of timed barium oesophagrams [31–33]. Some studies that compared PD and surgical esophagomyotomy concluded that surgical esophagomyotomy was better than PD [34–37], and others concluded that PD was better suited as the initial treatment of achalasia [38–40], and a study by West recommended surgical myotomy as over PD in the elderly [37]. Several articles published after 2006 reported comparable success rates for PD and LHM [33, 41, 42]. Postprocedure monitoring of the occurrence of adverse events such as fever, shortness of breath, chest pain, and subcutaneous emphysema for several hours after PD has been recommended. The most serious complication of PD is esophageal perforation, with a reported incidence of 0% to 8%, of cases [43]. If a perforation is suspected, a gastrografin study or barium esophagogram is recommended. If the recovery is uneventful, the patient can be given liquids and sent home.

Endoscopic injection of botulinum toxin (EBTI) is used to treat the pathophysiology of achalasia rather than by mechanically or surgically disrupting the sphincter muscle [28]. EBTI blocks the release of presynaptic acetylcholine at the neuromuscular junction of the LES to relax the muscle and relieve symptoms. Its advantages include simplicity, little trauma, few adverse reactions, and the tolerance of most patients. Its shortcomings are poor long-term efficacy, with sustained results for only several months [44]. Secondly, some patients with loss of the normal anatomic planes develop fibrosis at the level of the gastroesophageal junction, which is associated with increased incidence of mucosal perforation, difficulty in performing a subsequent myotomy, and a worse prognosis [32, 45]. Because of the shortcoming and low effectiveness of EBTI, only eight of the top 100 papers described the use of that therapy.

The use of LHM in achalasia was first reported by Shimi et al. in 1991 [46], and its use has expanded since then. It is more effective than other surgical treatments and has the longest remission time. The procedure can be performed in patients with no serious systemic disease, who have failed conservative treatment, experienced recurrences after repeated dilation, and have failed other treatments. Most experts recommend LHM as the first-line treatment for achalasia of the cardia. The fifth most cited article (428 citations) was published in 2011 by Boeckxstaens et al., and it compared the outcomes of LHM in 106 patients and PD in 95 patients [33].

The majority of the top 100 and the 482 articles reported the results of studies performed in the USA, and that is in line with similar studies of articles in the fields of digestive disease and emergency medicine [9, 13]. The preponderance of articles from the USA and other developed countries reflects the ample financial resources available to support research by the scientific community. It has also been reported that investigators in the USA tend to cite publications by other American authors [47, 48].

There are some study limitations. Although we searched data from the WoSCC, Scopus, and PubMed databases, we only included articles published in English, which may have lost sight of influential articles published in other languages and included in other databases. These limitations maybe have influenced the creation of this list of landmark articles. In addition, since the scope of our study was extremely wide, it was not able to perform a quantitative systematic review as Guzman-Ortiz et al. conducted [49], but we did a scoping review. In sum, achalasia research is extensive and it is constantly advancing. This review of papers of great significance and influence in the field of achalasia highlights some key topics and some major developments that have occurred over the last 25 years.

## 5. Conclusion

Our study offers a historical perspective on the progress of achalasia research and identified the articles that contributed the most to the prophylaxis and treatment of achalasia. The most cited articles, authors, journals, and title words are listed.

## Figures and Tables

**Table 1:** Inclusion criteria, exclusion criteria, and search strategy.

Inclusion criteria	(i) Language: English
	(ii) Data type: article, review, guideline, and consensus
	(iii) Keyword: achalasia
	(iv) Total citations: 30 or more times
	(v) Publish year: 1995–2020
Exclusion criteria	Publications that have not been based on topics of achalasia (Pseudoachalasia, Chagas disease)
Search strategy in PubMed	achalasia [Title/Abstract] AND (English [language]) AND ((“1995/01/01” [date-publication]: “2020/11/15” [date-publication]))
Search strategy in Scopus	TITLE-ABS-KEY (achalasia) AND PUBYEAR > 1994 AND (LIMIT-TO (LANGUAGE, “English”)
Search strategy in Web of Science	(TS = achalasia) AND (language = English)

**Table 2:** Characteristics of included scoping review studies.

Type of article	*N*	Percentage
Clinical research	357	74.1%
Review	87	18.0%
Basic science research	34	7.1%
Guideline and consensus	4	0.8%
*Study*	*N*	*Percentage*
Treatment	335	69.5%
Other	55	11.4%
Etiology	332	6.6%
All	31	6.4%
Diagnosis	19	3.9%
Classification	8	1.7%
Pathogenesis	2	0.5%

**Table 3:** The top-10 cited articles in Achalasia research.

Rank	First author	Journal	Title	Number of citations	Type of article	Study direction	Publish year
1	Inoue, H	Endoscopy	Peroral Endoscopic Myotomy (POEM) for Esophageal Achalasia	953	Clinical research	Treatment	2010
2	Kahrilas, PJ	Neurogastroenterol. Motil.	The Chicago Classification of Esophageal Motility Disorders, v3.0	894	Guideline and consensus	Classification	2015
3	Bredenoord, AJ	Neurogastroenterol. Motil.	Chicago Classification Criteria of Esophageal Motility Disorders Defined in High Resolution Esophageal Pressure Topography	541	Guideline and consensus	Classification	2012
4	Pandolfino, JE	Gastroenterology	Achalasia: A New Clinically Relevant Classification by High-Resolution Manometry	530	Clinical research	Classification	2008
5	Specler S.J.	Gastroenterology	Classification of Oesophageal Motility Abnormalities	514	Review	Classification	2001
6	Boeckxstaens, GE	N. Engl. J. Med.	Pneumatic Dilation versus Laparoscopic Heller's Myotomy for Idiopathic Achalasia	489	Clinical research	Treatment	2011
7	Campos, GM	Ann. Surg.	Endoscopic and Surgical Treatments for Achalasia A Systematic Review and Meta-Analysis	465	Review	Treatment	2009
8	Mittal R. K	N. Engl. J. Med.	The Esophagogastric Junction	460	Review	All	1997
9	Pasricha P.J.	N. Engl. J. Med.	Intrasphincteric Botulinum Toxin for the Treatment of Achalasia	458	Clinical research	Treatment	1995
10	Pasricha P.J.	Gastroenterology	Botulinum Toxin for Achalasia: Long-Term Outcome and Predictors of Response	332	Clinical research	Treatment	1996

**Table 4:** Journals with two or more articles of the top-100 cited articles.

Rank	Journal	No. of articles (%)	Total no. of citations	Average no. of citations per paper^✬^	CiteScore (2019)	IF (2019)	ESI (2019)	ES (2019)	AIS (2019)	Country
1	Annals of Surgery	15	3001	200	15	10.13	38.88	0.06148	3.163	USA
2	Gastroenterology	12	2738	208	24.7	17.373	70.38	0.10419	6.591	UK
3a	Gut	9	1995	222	32.2	19.819	62.44	0.07141	5.915	UK
3b	American Journal of Gastroenterology	9	1723	199	10.2	10.171	45.16	0.03757	3.763	Germany
4	Neurogastroenterology and Motility	6	2076	346	6.1	2.946	14.52	0.0118	0.897	UK
5	Surgical Endoscopy	5	727	145	6	3.149	13.26	0.03286	0.879	Germany
6a	Journal of the American College of Surgeons	4	613	153	7.8	4.59	22.65	0.02618	1.794	Netherlands
6b	Journal of Gastrointestinal Surgery	4	579	145	4.1	2.573	12.21	0.01502	0.892	Germany
6c	JAMA Surgery^∗^	4	535	134	14.7	13.625	28	0.03834	4.675	USA
7a	New England Journal of Medicine	3	1407	469	66.1	74.699	255.27	0.6618	31.294	USA
7b	Endoscopy	3	1395	465	7.4	7.341	21.37	0.01564	2.061	Germany
7c	Journal of the American Medical Association	3	535	178	26.3	45.54	140.26	0.29049	21.694	USA
8a	Journal of Clinical Gastroenterology	2	385	193	5	2.973	14.07	0.0093	0.913	USA
8b	Clinical Gastroenterology and Hepatology	2	307	154	9.5	8.549	29.31	0.03732	2.736	UK
8c	Digestive Diseases and Sciences	2	242	121	5.1	2.751	11.40	0.01951	0.792	USA
8d	World Journal of Surgery	2	237	119	4.5	2.234	12.37	0.02095	0.814	Germany
8e	Gastrointestinal Endoscopy	2	219	115	7.3	6.89	23.33	0.028	1.891	USA

IF: impact factor; ESI: Essential Science Indicators; ES: Eigenfactor™ score; AIS: Article Influence Score. ^✮^The average number of citations was rounded to the nearest integer number. ^∗^*JAMA Surgery*: the journal name was *Archives of Surgery* before 2015.

**Table 5:** Authors with two or more articles in top-100 cited articles.

Rank	Author	No. of articles	First	Second
1	Pandolfino, JE	6	5	1
2a	Zaninotto, G	5	4	1
2b	Inoue, H	5	3	2
2c	Kahrilas, PJ	5	3	2
2d	Annese, V	5	2	3
3a	Patti, MG	4	4	
3b	Vaezi, MF	4	3	1
3c	Richter, JE	4	1	3
3d	Costantini, M	4		4
4a	Pasricha, PJ	3	3	
4b	Eckardt, VF	3	2	1
4c	Bredenoord, AJ	3	1	2
4d	Ghosh, SK	3	1	2
4e	Khashab, MA	3	1	2
5a	Boeckxstaens, GE	2	2	
5b	Rohof, WO	2	2	
5c	Swanstrom, Lee L	2	2	
5d	Von Renteln, D	2	2	
5e	Fox, MR	2	1	1
5f	Richards, WO	2	1	1
5 g	Salvador, R	2	1	1
5 h	Torquati, A	2	1	1

**Table 6:** Countries of origin of the top-100 cited articles.

Rank	Country	No. of articles
1	USA	55
2	Italy	11
3	Germany	7
4	Japan	5
5a	Belgium	4
5b	China	4
5c	Netherlands	4
6a	Switzerland	2
6b	France	2
7a	Canada	1
7b	Chile	1
7c	Greece	1
7d	India	1
7e	Sweden	1
7f	United Kingdom	1

**Table 7:** Countries of origin of the top-500 cited articles.

Rank	Country	No. of articles
1	USA	217
2	Italy	51
3	Germany	32
4	China	30
5	Canada	24
6	Japan	21
7	Netherlands	12
8a	France	11
8b	Spain	11
9	Belgium	9
10	India	8
11	Australia	6
12a	Brazil	6
12b	Sweden	6
13a	Ireland	5
13b	United Kingdom	5
14	Turkey	4
15a	Iran	3
15b	Switzerland	3
15c	South Africa	3
16a	Argentina	2
16b	Chile	2
16c	Greece	2
16d	South Korea	2
17a	Czech Republic	1
17b	Egypt	1
17c	Mexico	1
17d	Pakistan	1
17e	Serbia	1
17f	Singapore	1

**Table 8:** Journals with four or more articles of the 482 articles which met all criteria.

Rank	Journal	No. of articles	Total citations	Average no. of citations per paper^✮^
1	American Journal of Gastroenterology	38	3563	94
2	Surgical Endoscopy	32	2137	67
3	Journal of Gastrointestinal Surgery	28	1832	65
4	Gastrointestinal Endoscopy	26	1517	58
5a	Digestive Diseases and Sciences	24	1435	60
5b	Annals of Surgery	24	3595	150
6	Gastroenterology	19	3159	166
7	Surgical Endoscopy and Other Interventional Techniques	18	871	48
8	Gut	17	2508	148
9	Diseases of the Esophagus	16	784	49
10	Neurogastroenterology and Motility	15	2463	164
11	Endoscopy	14	2132	152
12	Jama Surgery^∗^	12	974	81
13	World journal of gastroenterology	11	549	50
14a	Journal of Clinical Gastroenterology	10	749	75
14b	Alimentary Pharmacology and Therapeutics	10	584	58
15	Clinical Gastroenterology and Hepatology	9	666	74
16a	Journal of the American College of Surgeons	8	901	113
16b	Journal of Pediatric Surgery	8	346	43
17a	Gastrointestinal Endoscopy Clinics of North America	7	462	66
17b	Annals of Thoracic Surgery	7	357	51
18a	Surgery	5	313	63
18b	Journal of Pediatric Gastroenterology and Nutrition	5	239	48
18c	Digestive Endoscopy	5	245	49
18d	Journal of the American Medical Association	5	681	136
19a	World Journal of Surgery	4	387	97
19b	New England Journal of Medicine	4	1460	365
19c	Journal of Gastroenterology and Hepatology (Australia)	4	157	39
19d	American Journal of Surgery	4	252	63

^✮^The average number of citations was rounded to the nearest integer number. ^∗^*JAMA Surgery*: the journal name was *Archives of Surgery* before 2015.

## Data Availability

The data used to support the findings of this study are available from the corresponding author upon request.

## Supplementary Materials

Supplementary Materials Table S1: the eligible articles in achalasia research.

## References

[1] Finch J., Bell S., Bellingan L. (2013). Accessibility, sustainability, excellence: how to expand access to research publications. Executive summary. *International Microbiology*.

[2] Gyawali C. P. (2016). Achalasia: new perspectives on an old disease. *Neurogastroenterology and Motility*.

[3] Verlaan T., Rohof W. O., Bredenoord A. J., Eberl S., Rösch T., Fockens P. (2013). Effect of peroral endoscopic myotomy on esophagogastric junction physiology in patients with achalasia. *Gastrointestinal Endoscopy*.

[4] Arksey H., O’Malley L. (2005). Scoping studies: towards a methodological framework. *International Journal of Social Research Methodology*.

[5] Tricco A. C., Lillie E., Zarin W. (2018). PRISMA extension for scoping reviews (PRISMA-ScR): checklist and explanation. *Annals of Internal Medicine*.

[6] Roldan-Valadez E., Salazar-Ruiz S. Y., Ibarra-Contreras R., Rios C. (2019). Current concepts on bibliometrics: a brief review about impact factor, Eigenfactor score, CiteScore, SCImago Journal Rank, Source-Normalised Impact per Paper, H-index, and alternative metrics. *Irish Journal of Medical Science*.

[7] Zhao D., Strotmann A. (2015). Analysis and visualization of citation networks. *Synthesis Lectures on Information Concepts Retrieval & Services*.

[8] van Eck N. J., Waltman L. (2010). Software survey: VOSviewer, a computer program for bibliometric mapping. *Scientometrics*.

[9] Tang X., Gong W., Yuan F. (2016). Top-cited articles in digestive system disease from 1950 to 2013. *Journal of Gastroenterology and Hepatology*.

[10] Hennessey K., Afshar K., Macneily A. E. (2009). The top 100 cited articles in urology. *Canadian Urological Association Journal*.

[11] Mazhari S. (2013). The 100 top-cited articles published in psychiatric journals. *Journal of Psychiatric Practice*.

[12] Ohba N., Nakao K., Isashiki Y., Ohba A. (2007). The 100 most frequently cited articles in ophthalmology journals. *Archives of Ophthalmology*.

[13] Tsai Y. L., Lee C. C., Chen S. C., Yen Z. S. (2006). Top-cited articles in emergency medicine. *The American Journal of Emergency Medicine*.

[14] Lefaivre K. A., Shadgan B., O’Brien P. J. (2011). 100 most cited articles in orthopaedic surgery. *Clinical Orthopaedics and Related Research*.

[15] Mayberry J. F., Mayell M. J. (1988). Epidemiological study of achalasia in children. *Gut*.

[16] Marlais M., Fishman J. R., Fell J. M., Haddad M. J., Rawat D. J. (2011). UK incidence of achalasia: an 11-year national epidemiological study. *Archives of Disease in Childhood*.

[17] Spechler S. J., Castell D. O. (2001). Classification of oesophageal motility abnormalities. *Gut*.

[18] Pandolfino J. E., Ghosh S. K., Rice J., Clarke J. O., Kwiatek M. A., Kahrilas P. J. (2008). Classifying esophageal motility by pressure topography characteristics: a study of 400 patients and 75 controls. *The American Journal of Gastroenterology*.

[19] Bredenoord A. J., Fox M., Kahrilas P. J., Pandolfino J. E., Schwizer W., Smout A. J. P. M. (2012). Chicago classification criteria of esophageal motility disorders defined in high resolution esophageal pressure topography. *Neurogastroenterology & Motility*.

[20] Boeckxstaens G., Zaninotto G. (2012). Achalasia and esophago-gastric junction outflow obstruction: focus on the subtypes. *Neurogastroenterology and Motility*.

[21] Kahrilas P. J., Bredenoord A. J., Fox M. (2015). The Chicago classification of esophageal motility disorders, v3.0. *Neurogastroenterology and Motility*.

[22] Salvador R., Voltarel G., Savarino E. (2018). The natural history of achalasia: evidence of a continuum-“The evolutive pattern theory”. *Digestive and Liver Disease*.

[23] Schlottmann F., Patti M. G. (2018). Esophageal achalasia: current diagnosis and treatment. *Expert Review of Gastroenterology & Hepatology*.

[24] Salvador R., Constantini M., Zaninotto G. (2010). The preoperative manometric pattern predicts the outcome of surgical treatment for esophageal achalasia. *Journal of Gastrointestinal Surgery*.

[25] Pratap N., Reddy D. N. (2011). Can achalasia subtyping by high-resolution manometry predict the therapeutic outcome of pneumatic balloon dilatation? : author’s reply. *J Neurogastroenterol Motil.*.

[26] Pandolfino J. E., Kwiatek M. A., Nealis T., Bulsiewicz W., Post J., Kahrilas P. J. (2008). Achalasia: a new clinically relevant classification by high-resolution manometry. *Gastroenterology*.

[27] Inoue H., Minami H., Kobayashi Y. (2010). Peroral endoscopic myotomy (POEM) for esophageal achalasia. *Endoscopy*.

[28] Swanstrom L. L., Kurian A., Dunst C. M., Sharata A., Bhayani N., Rieder E. (2012). Long-term outcomes of an endoscopic myotomy for achalasia: the POEM procedure. *Annals of Surgery*.

[29] Kumbhari V., Tieu A. H., Onimaru M. (2015). Peroral endoscopic myotomy (POEM) vs laparoscopic Heller myotomy (LHM) for the treatment of type III achalasia in 75 patients: a multicenter comparative study. *Endosc Int Open.*.

[30] Khashab M. A., Messallam A. A., Onimaru M. (2015). International multi-center experience with peroral endoscopic myotomy for the treatment of spastic esophageal disorders refractory to medical therapy (with video). *Gastrointestinal Endoscopy*.

[31] Vantrappen G., Hellemans J., Deloof W., Valembois P., Vandenbroucke J. (1971). Treatment of achalasia with pneumatic dilatations. *Gut*.

[32] Eckardt V. F., Aignherr C., Bernhard G. (1992). Predictors of outcome in patients with achalasia treated by pneumatic dilation. *Gastroenterology*.

[33] Boeckxstaens G. E., Annese V. (2011). Pneumatic dilation versus laparoscopic Heller’s myotomy for idiopathic achalasia. *The New England Journal of Medicine*.

[34] Csendes A., Braghetto I., Henríquez A., Cortés C. (1989). Late results of a prospective randomised study comparing forceful dilatation and oesophagomyotomy in patients with achalasia. *Gut*.

[35] Spiess A. E., Kahrilas P. J. (1998). Treating achalasia: from whalebone to laparoscope. *Journal of the American Medical Association*.

[36] Okike N., Payne W. S., Neufeld D. M., Bernatz P. E., Pairolero P. C., Sanderson D. R. (1979). Esophagomyotomy versus forceful dilation for achalasia of the esophagus: results in 899 patients. *The Annals of Thoracic Surgery*.

[37] West R. L., Hirsch D. P., Bartelsman J. F. (2002). Long term results of pneumatic dilation in achalasia followed for more than 5 years. *The American Journal of Gastroenterology*.

[38] Parkman H. P., Reynolds J. C., Ouyang A., Rosato E. F., Eisenberg J. M., Cohen S. (1993). Pneumatic dilatation or esophagomyotomy treatment for idiopathic achalasia: clinical outcomes and cost analysis. *Digestive Diseases and Sciences*.

[39] Kadakia S. C., Wong R. K. (1993). Graded pneumatic dilation using Rigiflex achalasia dilators in patients with primary esophageal achalasia. *The American Journal of Gastroenterology*.

[40] Fellows I. W., Ogilvie A. L., Atkinson M. (1983). Pneumatic dilatation in achalasia. *Gut*.

[41] Vela M. F., Richter J. E., Khandwala F. (2006). The long-term efficacy of pneumatic dilatation and Heller myotomy for the treatment of achalasia. *Clinical Gastroenterology and Hepatology*.

[42] Moonen A., Annese V., Belmans A., Bredenoord A. J. (2016). Long-term results of the European achalasia trial: a multicentre randomised controlled trial comparing pneumatic dilation versus laparoscopic Heller myotomy. *Gut*.

[43] Campos G. M., Vittinghoff E., Rabl C. (2009). Endoscopic and surgical treatments for achalasia: a systematic review and meta-analysis. *Annals of Surgery*.

[44] Patti M. G., Feo C. V., Arcerito M. (1999). Effects of previous treatment on results of laparoscopic Heller myotomy for achalasia. *Digestive Diseases and Sciences*.

[45] Smith C. D., Stival A., Howell D. L., Swafford V. (2006). Endoscopic therapy for achalasia before Heller myotomy results in worse outcomes than Heller myotomy alone. *Annals of Surgery*.

[46] Shimi S., Nathanson L. K., Cuschieri A. (1991). Laparoscopic cardiomyotomy for achalasia. *Journal of the Royal College of Surgeons of Edinburgh*.

[47] Campbell F. M. (1990). National bias: a comparison of citation practices by health professionals. *Bulletin of the Medical Library Association*.

[48] Lin A. M. (1998). US and non-US submissions. *JAMA*.

[49] Guzman-Ortiz E., Bueno-Hernandez N., Melendez-Mier G., Roldan-Valadez E. (2020). Quantitative systematic review: methods used for the in vivo measurement of body composition in pregnancy. *Journal of Advanced Nursing*.

