# Supplementary material for: Scoping Review and Bibliometric Analysis of the Most Influential Publications in Achalasia Research from 1995 to 2020
Source: Biomed Res Int. 2021 Feb 4;2021:8836395. doi: 10.1155/2021/8836395 (PMC7884120; doi:10.1155/2021/8836395)
Supplement: Supplementary Materials — Table S1: the eligible articles in achalasia research. [file 8836395.f1.docx]

| Rank | First author | Title | Journal | Study direction | Type of article | Publish  year | No. of  citations |
| --- | --- | --- | --- | --- | --- | --- | --- |
| 1 | Inoue H. | Peroral endoscopic myotomy (POEM) for esophageal achalasia | Endoscopy | Treatment | clinical research | 2010 | 953 |
| 2 | Kahrilas P.J. | The Chicago Classification of esophageal motility disorders, v3.0 | Neurogastroenterology and Motility | Classification | guideline and consensus | 2015 | 894 |
| 3 | Bredenoord A.J. | Chicago classification criteria of esophageal motility disorders defined in high resolution esophageal pressure topography | Neurogastroenterology and Motility | Classification | guideline and consensus | 2012 | 541 |
| 4 | Pandolfino J.E. | Achalasia: A New Clinically Relevant Classification by High-Resolution Manometry | Gastroenterology | Classification | clinical research | 2008 | 530 |
| 5 | Specler S.J. | Classification of oesophageal motility abnormalities | Gut | Classification | review | 2001 | 514 |
| 6 | Boeckxstaens G.E. | Pneumatic dilation versus laparoscopic heller's myotomy for idiopathic achalasia | New England Journal of Medicine | Treatment | clinical research | 2011 | 489 |
| 7 | Campos G.M. | Endoscopic and surgical treatments for achalasia: A systematic review and meta-analysis | Annals of Surgery | Treatment | review | 2009 | 465 |
| 8 | Mittal R.K | The esophagogastric junction | New England Journal of Medicine | All | review | 1997 | 460 |
| 9 | Pasricha P.J. | Intrasphincteric botulinum toxin for the treatment of achalasia | New England Journal of Medicine | Treatment | clinical research | 1995 | 458 |
| 10 | Pasricha P.J. | Botulinum toxin for achalasia: Long-term outcome and predictors of response | Gastroenterology | Treatment | clinical research | 1996 | 332 |
| 11 | Pasricha P.J. | Submucosal endoscopic esophageal myotomy: A novel experimental approach for the treatment of achalasia | Endoscopy | Treatment | clinical research | 2007 | 324 |
| 12 | Richards, WO | Heller myotomy versus Heller myotomy with Dor fundoplication for achalasia - A prospective randomized double-blind clinical trial | Annals of Surgery | Treatment | clinical research | 2004 | 318 |
| 13 | Pandolfino J.E. | Classifying esophageal motility by pressure topography characteristics: A study of 400 patients and 75 controls | American Journal of Gastroenterology | Classification | clinical research | 2008 | 286 |
| 14 | Von Renteln D. | Peroral endoscopic myotomy for the treatment of achalasia: A prospective single center study | American Journal of Gastroenterology | Treatment | clinical research | 2012 | 267 |
| 15 | Boeckxstaens G.E. | Achalasia | The Lancet | All | review | 2014 | 259 |
| 16 | Fox M.R. | Oesophageal high-resolution manometry: Moving from research into clinical practice | Gut | Classification | review | 2008 | 257 |
| 17 | Park W. | Etiology and pathogenesis of achalasia: The current understanding | American Journal of Gastroenterology | All | review | 2005 | 257 |
| 18 | Patti, MG; | Minimally invasive surgery for achalasia - An 8-year experience with 168 patients | Annals of Surgery | Treatment | clinical research | 1999 | 255 |
| 19 | Spiess A.E. | Treating achalasia: From whalebone to laparoscope | Journal of the American Medical Association | Treatment | review | 1998 | 242 |
| 20 | Inoue H. | Per-Oral Endoscopic Myotomy: A Series of 500 Patients | Journal of the American College of Surgeons | Treatment | review | 2015 | 241 |
| 21 | Von Renteln D. | Peroral endoscopic myotomy for the treatment of achalasia: An international prospective multicenter study | Gastroenterology | Treatment | clinical research | 2013 | 235 |
| 22 | Vaezi M.F. | Botulinum toxin versus pneumatic dilatation in the treatment of achalasia: A randomized trial | Gut | Treatment | clinical research | 1999 | 234 |
| 23 | Pandolfino J.E. | AGA technical review on the clinical use of esophageal manometry | Gastroenterology | All | review | 2005 | 230 |
| 24 | Pandolfino J.E. | High-resolution manometry in clinical practice: Utilizing pressure topography to classify oesophageal motility abnormalities | Neurogastroenterology and Motility | Classification | review | 2009 | 221 |
| 25 | Richter J.E. | Current therapies for achalasia: Comparison and efficacy | Journal of Clinical Gastroenterology | Treatment | review | 1998 | 221 |
| 26 | Eckardt V.F. | Pneumatic dilation for achalasia: Late results of a prospective follow up investigation | Gut | Treatment | clinical research | 2004 | 219 |
| 27 | Rohof W.O. | Outcomes of treatment for achalasia depend on manometric subtype | Gastroenterology | Treatment | clinical research | 2013 | 218 |
| 28 | Goldblum J.R | Histopathologic features in esophagomyotomy specimens from patients with achalasia | Gastroenterology | Other | clinical research | 1996 | 216 |
| 29 | Hunter J.G. | Laparoscopic heller myotomy and fundoplication for achalasia | Annals of Surgery | Treatment | clinical research | 1997 | 212 |
| 30 | Swanstrom, Lee L. | Long-Term Outcomes of an Endoscopic Myotomy for Achalasia The POEM Procedure | Annals of Surgery | Treatment | clinical research | 2012 | 210 |
| 31 | Ghosh S.K. | Impaired deglutitive EGJ relaxation in clinical esophageal manometry: A quantitative analysis of 400 patients and 75 controls | American Journal of Physiology - Gastrointestinal and Liver Physiology | Diagnosis | clinical research | 2007 | 202 |
| 32 | Bhayani N.H. | A comparative study on comprehensive, objective outcomes of laparoscopic heller myotomy with Per-Oral Endoscopic Myotomy (POEM) for achalasia | Annals of Surgery | Treatment | clinical research | 2014 | 192 |
| 33 | Zaninotto G. | Four hundred laparoscopic myotomies for esophageal achalasia a single centre experience | Annals of Surgery | Treatment | clinical research | 2008 | 191 |
| 34 | Vaezi M.F. | Timed barium oesophagram: Better predictor of long term success after pneumatic dilation in achalasia than symptom assessment | Gut | Diagnosis | clinical research | 2002 | 188 |
| 35 | Vela M.F. | The Long-term Efficacy of Pneumatic Dilatation and Heller Myotomy for the Treatment of Achalasia | Clinical Gastroenterology and Hepatology | Treatment | clinical research | 2006 | 187 |
| 36 | Clouse R.E. | Application of topographical methods to clinical esophageal manometry | American Journal of Gastroenterology | All | clinical research | 2000 | 184 |
| 37 | Oelschlager, BK | Improved outcome after extended gastric myotomy for achalasia | Archives of Surgery | Treatment | clinical research | 2003 | 182 |
| 38 | Vaezi M.F. | Diagnosis and management of achalasia | American Journal of Gastroenterology | Diagnosis | review | 1999 | 182 |
| 39 | Hungness E.S. | Comparison of Perioperative Outcomes Between Peroral Esophageal Myotomy (POEM) and Laparoscopic Heller Myotomy | Journal of Gastrointestinal Surgery | Treatment | clinical research | 2013 | 180 |
| 40 | Zaninotto G. | Randomized Controlled Trial of Botulinum Toxin Versus Laparoscopic Heller Myotomy for Esophageal Achalasia | Annals of Surgery | Treatment | clinical research | 2004 | 179 |
| 41 | Annese V. | A multicentre randomised study of intrasphincteric botulinum toxin in patients with oesophageal achalasia | Gut | Treatment | clinical research | 2000 | 175 |
| 42 | Rawlings A. | Laparoscopic Dor versus Toupet fundoplication following Heller myotomy for achalasia: Results of a multicenter, prospective, randomized-controlled trial | Surgical Endoscopy | Treatment | clinical research | 2012 | 171 |
| 43 | Smith C.D. | Endoscopic therapy for achalasia before heller myotomy results in worse outcomes than heller myotomy alone | Annals of Surgery | Treatment | clinical research | 2006 | 169 |
| 44 | Rebecchi F. | Randomized controlled trial of laparoscopic heller myotomy plus dor fundoplication versus nissen fundoplication for achalasia long-term results | Annals of Surgery | Treatment | clinical research | 2008 | 168 |
| 45 | Stavropoulos S.N. | The international per oral endoscopic myotomy survey (IPOEMS): A snapshot of the global POEM experience | Surgical Endoscopy | Treatment | Clinical research | 2013 | 166 |
| 46 | Annese V. | Controlled trial of botulinum toxin injection versus placebo and pneumatic dilation in achalasia | Gastroenterology | Treatment | clinical research | 1996 | 165 |
| 47 | Kahrilas, Peter J. | Esophageal motility disorders in terms of pressure topography - The Chicago classification | Journal of Clinical Gastroenterology | Classification | review | 2008 | 164 |
| 48 | Mayberry J.F. | Epidemiology and demographics of achalasia | Gastrointestinal Endoscopy Clinics of North America | Other | review | 2001 | 164 |
| 49 | Fox, M | High-resolution manometry predicts the success of oesophageal bolus transport and identifies clinically important abnormalities not detected by conventional manometry | Neurogastroenterology and Motility | Diagnosis | clinical research | 2004 | 161 |
| 50 | West R.L. | Long term results of pneumatic dilation in achalasia followed for more than 5 years | American Journal of Gastroenterology | Treatment | clinical research | 2002 | 160 |
| 51 | Salvador R. | The Preoperative Manometric Pattern Predicts the Outcome of Surgical Treatment for Esophageal Achalasia | Journal of Gastrointestinal Surgery | Treatment | clinical research | 2010 | 159 |
| 52 | Rohof W.O. | Efficacy of treatment for patients with achalasia depends on the distensibility of the esophagogastric junction | Gastroenterology | Treatment | clinical research | 2012 | 159 |
| 53 | Zaninotto G. | Etiology, diagnosis, and treatment of failures after laparoscopic Heller myotomy for achalasia | Annals of Surgery | All | clinical research | 2002 | 154 |
| 54 | Moonen A. | Long-term results of the European Achalasia trial: A multicentre randomised Controlled trial comparing pneumatic dilation versus laparoscopic Heller myotomy | Gut | Treatment | clinical research | 2016 | 152 |
| 55 | Ren Z | Perioperative management and treatment for complications during and after peroral endoscopic myotomy (POEM) for esophageal achalasia (EA) (data from 119 cases) | Surgical Endoscopy | Treatment | clinical research | 2012 | 151 |
| 56 | Sandler R.S. | The risk of esophageal cancer in patients with achalasia: A population- based study | Journal of the American Medical Association | Other | clinical research | 1995 | 147 |
| 57 | Patti M.G. | Impact of minimally invasive surgery on the treatment of esophageal achalasia: A decade of change | Journal of the American College of Surgeons | Treatment | clinical research | 2003 | 146 |
| 58 | Pandolfino J.E. | Achalasia: A systematic review | JAMA - Journal of the American Medical Association | All | review | 2015 | 146 |
| 59 | Csendes A. | Very late results of esophagomyotomy for patients with achalasia: Clinical, endoscopic, histologic, manometric, and acid reflux studies in 67 patients for a mean follow-up of 190 months | Annals of Surgery | Treatment | clinical research | 2006 | 145 |
| 60 | Sadowski D.C. | Achalasia: Incidence, prevalence and survival. A population-based study | Neurogastroenterology and Motility | Other | clinical research | 2010 | 144 |
| 61 | Bortolotti M. | Effects of sildenafil on esophageal motility of patients with idiopathic achalasia | Gastroenterology | Treatment | clinical research | 2000 | 143 |
| 62 | Francis D.L. | Achalasia: Update on the disease and its treatment | Gastroenterology | All | review | 2010 | 141 |
| 63 | Ancona E. | Esophageal achalasia: Laparoscopic versus conventional open heller-dor operation | The American Journal of Surgery | Treatment | clinical research | 1995 | 139 |
| 64 | Zerbib F. | Repeated pneumatic dilations as long-term maintenance therapy for esophageal achalasia | American Journal of Gastroenterology | Treatment | clinical research | 2006 | 136 |
| 65 | Sharp K.W. | 100 Consecutive minimally invasive heller myotomies: Lessons learned | Annals of Surgery | Treatment | clinical research | 2002 | 135 |
| 66 | Parkman H.P. | Symptomatic improvement in achalasia after botulinum toxin injection of the lower esophageal sphincter | American Journal of Gastroenterology | Treatment | clinical research | 1996 | 131 |
| 67 | Horgan S. | Does botulinum toxin injection make esophagomyotomy a more difficult operation? | Surgical Endoscopy | Treatment | clinical research | 1999 | 131 |
| 68 | Clark S.B. | The nature of the myenteric infiltrate in achalasia: An immunohistochemical analysis | American Journal of Surgical Pathology | Other | basic science research | 2000 | 130 |
| 69 | Hirano I. | Alimentary tract: Manometric heterogeneity in patients with idiopathic achalasia | Gastroenterology | Other | clinical research | 2001 | 133 |
| 70 | Sivarao D.V. | Lower esophageal sphincter is achalasic in nNOS-/- and hypotensive in W/Wv mutant mice | Gastroenterology | Etiology | basic science research | 2001 | 130 |
| 71 | Devaney E.J. | Esophagectomy for achalasia: Patient selection and clinical experience | Annals of Thoracic Surgery | Treatment | clinical research | 2001 | 129 |
| 72 | Cuillière C. | Achalasia: Outcome of patients treated with intrasphincteric injection of botulinum toxin | Gut | Treatment | clinical research | 1997 | 129 |
| 73 | De Oliveira J.M.A. | Timed barium swallow: A simple technique for evaluating esophageal emptying in patients with achalasia | American Journal of Roentgenology | Diagnosis | review | 1997 | 128 |
| 74 | Costamagna G. | Peroral endoscopic myotomy (POEM) for oesophageal achalasia: Preliminary results in humans | Digestive and Liver Disease | Treatment | clinical research | 2012 | 128 |
| 75 | Patti M.G. | Laparoscopic Heller myotomy and Dor fundoplication for achalasia: Analysis of successes and failures | Archives of Surgery | Treatment | clinical research | 2001 | 127 |
| 76 | Patti M.G. | Effects of previous treatment on results of laparoscopic Heller myotomy for achalasia | Digestive Diseases and Sciences | Treatment | clinical research | 1999 | 127 |
| 77 | Werner Y.B. | Clinical response to peroral endoscopic myotomy in patients with idiopathic achalasia at a minimum follow-up of 2 years | Gut | Treatment | clinical research | 2016 | 127 |
| 78 | Inoue H. | Peroral Endoscopic Myotomy for Esophageal Achalasia: Technique, Indication, and Outcomes | Thoracic Surgery Clinics | Treatment | review | 2011 | 126 |
| 79 | Zaninotto G. | Treatment of Esophageal Achalasia with Laparoscopic Heller Myotomy and Dor Partial Anterior Fundoplication: Prospective Evaluation of 100 Consecutive Patients | Journal of Gastrointestinal Surgery | Treatment | clinical research | 2000 | 125 |
| 80 | Kostic S. | Pneumatic dilatation or laparoscopic cardiomyotomy in the management of newly diagnosed idiopathic achalasia: Results of a randomized controlled trial | World Journal of Surgery | Treatment | clinical research | 2007 | 124 |
| 81 | Hulselmans M | Long-term Outcome of Pneumatic Dilation in the Treatment of Achalasia | Clinical Gastroenterology and Hepatology | Treatment | clinical research | 2010 | 120 |
| 82 | Karamanolis G. | Long-term outcome of pneumatic dilation in the treatment of achalasia | American Journal of Gastroenterology | Treatment | clinical research | 2005 | 120 |
| 83 | Zhou P.H. | Peroral endoscopic remyotomy for failed Heller myotomy: A prospective single-center study | Endoscopy | Treatment | clinical research | 2013 | 118 |
| 84 | Swanström L.L. | A stepwise approach and early clinical experience in peroral endoscopic myotomy for the treatment of achalasia and esophageal motility disorders | Journal of the American College of Surgeons | Treatment | clinical research | 2011 | 116 |
| 85 | Sharata A.M. | Peroral Endoscopic Myotomy (POEM) for Esophageal Primary Motility Disorders: Analysis of 100 Consecutive Patients | Journal of Gastrointestinal Surgery | Treatment | clinical research | 2014 | 115 |
| 86 | Wang L. | Meta-analysis of randomized and controlled treatment trials for achalasia | Digestive Diseases and Sciences | Treatment | review | 2009 | 115 |
| 87 | Raiser F. | Heller myotomy via minimal-access surgery: An evaluation of antireflux procedures | Archives of Surgery | Treatment | clinical research | 1996 | 115 |
| 88 | Pratap N. | Achalasia cardia subtyping by high-resolution manometry predicts the therapeutic outcome of pneumatic balloon dilatation | Journal of Neurogastroenterology and Motility | Treatment | clinical research | 2011 | 115 |
| 89 | Brücher B.L.D.M. | Achalasia and esophageal cancer: Incidence, prevalence, and prognosis | World Journal of Surgery | Other | clinical research | 2001 | 113 |
| 90 | Khajanchee Y.S. | Laparoscopic Heller myotomy with Toupet fundoplication: Outcomes predictors in 121 consecutive patients | Archives of Surgery | Treatment | clinical research | 2005 | 111 |
| 91 | Pohl D. | Achalasia: An overview of diagnosis and treatment | Journal of Gastrointestinal and Liver Diseases | All | review | 2007 | 111 |
| 92 | Kurian, Ashwin A. | Peroral endoscopic esophageal myotomy: defining the learning curve | Gastrointestinal Endoscopy | Treatment | clinical research | 2013 | 110 |
| 93 | Onimaru M. | Peroral endoscopic myotomy is a viable option for failed surgical esophagocardiomyotomy instead of redo surgical Heller myotomy: A single center prospective study | Journal of the American College of Surgeons | Treatment | clinical research | 2013 | 110 |
| 94 | Eckardt A.J. | Treatment and surveillance strategies in achalasia: An update | Nature Reviews Gastroenterology and Hepatology | Treatment | review | 2011 | 109 |
| 95 | Khashab M.A. | International multicenter experience with peroral endoscopic myotomy for the treatment of spastic esophageal disorders refractory to medical therapy (with video) | Gastrointestinal Endoscopy | Treatment | clinical research | 2015 | 109 |
| 96 | Wright A.S. | Long-term outcomes confirm the superior efficacy of extended Heller myotomy with Toupet fundoplication for achalasia | Surgical Endoscopy and Other Interventional Techniques | Treatment | clinical research | 2007 | 108 |
| 97 | Taketomi T. | Loss of mammalian Sprouty2 leads to enteric neuronal hyperplasia and esophageal achalasia | Nature Neuroscience | Etiology | basic science research | 2005 | 107 |
| 98 | Kahrilas P.J. | High-Resolution Manometry and Impedance-pH/Manometry: Valuable Tools in Clinical and Investigational Esophagology | Gastroenterology | Diagnosis | review | 2008 | 106 |
| 99 | Schlottmann F. | Laparoscopic Heller Myotomy Versus Peroral Endoscopic Myotomy (POEM) for Achalasia: A Systematic Review and Meta-analysis | Annals of Surgery | Treatment | review | 2018 | 105 |
| 100 | Torquati A. | Laparoscopic myotomy for achalasia: Predictors of successful outcome after 200 cases | Annals of Surgery | Treatment | clinical research | 2006 | 103 |
| 101 | Eckardt V.F. | Chest pain in achalasia: Patient characteristics and clinical course | Gastroenterology | Other | clinical research | 1999 | 103 |
| 102 | Li Q.-L | Peroral endoscopic myotomy for the treatment of achalasia: A Clinical comparative study of endoscopic full-thickness and circular muscle myotomy | Journal of the American College of Surgeons | Treatment | clinical research | 2013 | 101 |
| 103 | Ujiki M.B. | Peroral endoscopic myotomy: A short-term comparison with the standard laparoscopic approach | Surgery (United States) | Treatment | clinical research | 2013 | 100 |
| 104 | Richter J.E. | Management of achalasia: Surgery or pneumatic dilation | Gut | Treatment | Review | 2011 | 99 |
| 105 | Talukdar R. | Efficacy of peroral endoscopic myotomy (POEM) in the treatment of achalasia: a systematic review and meta-analysis | Surgical Endoscopy | Treatment | Review | 2015 | 99 |
| 106 | Streitz Jr. J.M. | Achalasia and squamous cell carcinoma of the esophagus: Analysis of 241 patients | The Annals of Thoracic Surgery | Other | Review | 1995 | 99 |
| 107 | Mikaeli J. | Randomized controlled trial comparing botulinum toxin injection to pneumatic dilatation for the treatment of achalasia | Alimentary Pharmacology and Therapeutics | Treatment | clinical research | 2001 | 98 |
| 108 | Stavropoulos S.N. | Per-oral endoscopic myotomy white paper summary | Gastrointestinal Endoscopy | Treatment | review | 2014 | 98 |
| 109 | Ghoshal U.C. | Long-term follow-up after pneumatic dilation for achalasia cardia: Factors associated with treatment failure and recurrence | American Journal of Gastroenterology | Treatment | clinical research | 2004 | 98 |
| 110 | Farhoomand K. | Predictors of outcome of pneumatic dilation in achalasia | Clinical Gastroenterology and Hepatology | Treatment | clinical research | 2004 | 96 |
| 111 | Katz P.O. | Pneumatic dilatation is effective long-term treatment for achalasia | Digestive Diseases and Sciences | Treatment | clinical research | 1998 | 96 |
| 112 | Allescher, HD | Treatment of achalasia: Botulinum toxin injection vs. pneumatic balloon dilation. A prospective study with long-term follow-up | ENDOSCOPY | Treatment | clinical research | 2001 | 95 |
| 113 | Leeuwenburgh I. | Long-term esophageal cancer risk in patients with primary achalasia: A prospective study | American Journal of Gastroenterology | Treatment | clinical research | 2010 | 95 |
| 114 | Kumbhari, Vivek | Peroral endoscopic myotomy (POEM) vs laparoscopic Heller myotomy (LHM) for the treatment of Type III achalasia in 75 patients: a multicenter comparative study | ENDOSCOPY INTERNATIONAL OPEN | Treatment | clinical research | 2015 | 94 |
| 115 | Stewart K.C. | Thoracoscopic versus laparoscopic modified Heller myotomy for achalasia: Efficacy and safety in 87 patients | Journal of the American College of Surgeons | Treatment | clinical research | 1999 | 94 |
| 116 | Horgan S. | Robotic-assisted Heller myotomy versus laparoscopic Heller myotomy for the treatment of esophageal achalasia: Multicenter study | Journal of Gastrointestinal Surgery | Treatment | clinical research | 2005 | 94 |
| 117 | Lopushinsky S.R. | Pneumatic dilatation and surgical myotomy for achalasia | Journal of the American Medical Association | Treatment | clinical research | 2006 | 94 |
| 118 | Vaezi M.F. | Assessment of esophageal emptying post-pneumatic dilation: Use of the timed barium esophagram | American Journal of Gastroenterology | Treatment | clinical research | 1999 | 94 |
| 119 | Kahrilas P.J. | The spectrum of achalasia: Lessons from studies of pathophysiology and high-resolution manometry | Gastroenterology | Etiology | Review | 2013 | 93 |
| 120 | Pandolfino J.E. | Distensibility of the esophagogastric junction assessed with the functional lumen imaging probe (FLIP™) in achalasia patients | Neurogastroenterology and Motility | Treatment | clinical research | 2013 | 93 |
| 121 | Moses P.L. | Antineuronal antibodies in idiopathic achalasia and gastro-oesophageal reflux disease | Gut | Etiology | basic science research | 2003 | 93 |
| 122 | Swanstrom L.L. | Laparoscopic esophagomyotomy for achalasia | Surgical Endoscopy | Treatment | clinical research | 1995 | 92 |
| 123 | O'Neill O.M. | Achalasia: A review of clinical diagnosis, epidemiology, treatment and outcomes | World Journal of Gastroenterology | All | review | 2013 | 92 |
| 124 | Ruiz-de-León A. | Myenteric antiplexus antibodies and class II HLA in achalasia | Digestive Diseases and Sciences | Etiology | basic science research | 2002 | 91 |
| 125 | Muehldorfer S.M. | Esophageal achalasia: Intrasphincteric injection of botulinum toxin A versus balloon dilation | Endoscopy | Treatment | clinical research | 1999 | 91 |
| 126 | Fisichella P.M. | Clinical, radiological, and manometric profile in 145 patients with untreated achalasia | World Journal of Surgery | Other | clinical research | 2008 | 90 |
| 127 | Akintoye E. | Peroral endoscopic myotomy: a meta-analysis | Endoscopy | Treatment | review | 2016 | 89 |
| 128 | Chiu P.W.Y. | Peroral endoscopic myotomy for treatment of achalasia: From bench to bedside (with video) | Gastrointestinal Endoscopy | Treatment | clinical research | 2013 | 89 |
| 129 | Sharata A. | Peroral Endoscopic Myotomy (POEM) Is Safe and Effective in the Setting of Prior Endoscopic Intervention | Journal of Gastrointestinal Surgery | Treatment | clinical research | 2013 | 89 |
| 130 | Vela M.F. | Complexities of managing achalasia at a tertiary referral center: Use of pneumatic dilatation, heller myotomy, and botulinum toxin injection | American Journal of Gastroenterology | Treatment | clinical research | 2004 | 89 |
| 131 | Verne G.N. | Anti-myenteric neuronal antibodies in patients with achalasia. A prospective study | Digestive Diseases and Sciences | Etiology | basic science research | 1997 | 89 |
| 132 | Falkenback D. | Heller's esophagomyotomy with or without a 360° floppy Nissen fundoplication for achalasia. Long-term results from a prospective randomized study | Diseases of the Esophagus | Treatment | clinical research | 2003 | 88 |
| 133 | Familiari P. | Peroral endoscopic myotomy for esophageal Achalasia: Outcomes of the first 100 patients with short-term follow-up | Annals of Surgery | Treatment | clinical research | 2016 | 87 |
| 134 | Rossetti G. | A total fundoplication is not an obstacle to esophageal emptying after heller myotomy for achalasia: Results of a long-term follow up | Annals of Surgery | Treatment | clinical research | 2005 | 87 |
| 135 | Lynch K.L. | Major complications of pneumatic dilation and heller myotomy for Achalasia: Single-center experience and systematic review of the literature | American Journal of Gastroenterology | Other | Review | 2012 | 86 |
| 136 | Kolbasnik J. | Long-term efficacy of Botulinum toxin in classical achalasia: A prospective study | American Journal of Gastroenterology | Treatment | clinical research | 1999 | 86 |
| 137 | Yaghoobi M. | Laparoscopic Heller's myotomy versus pneumatic dilation in the treatment of idiopathic achalasia: A meta-analysis of randomized, controlled trials | Gastrointestinal Endoscopy | Treatment | review | 2013 | 82 |
| 138 | Spechler S.J. | Heartburn in patients with achalasia | Gut | Other | clinical research | 1995 | 82 |
| 139 | Costantini M. | The laparoscopic Heller-Dor operation remains an effective treatment for esophageal achalasia at a minimum 6-year follow-up | Surgical Endoscopy and Other Interventional Techniques | Treatment | clinical research | 2005 | 81 |
| 140 | Familiari P. | Peroral Endoscopic Myotomy for Esophageal Achalasia Outcomes of the First 100 Patients With Short-term Follow-up | ANNALS OF SURGERY | Treatment | clinical research | 2016 | 80 |
| 141 | Teitelbaum E.N. | Symptomatic and physiologic outcomes one year after peroral esophageal myotomy (POEM) for treatment of achalasia | Surgical Endoscopy | Treatment | clinical research | 2014 | 80 |
| 142 | De Giorgio R. | Esophageal and gastric nitric oxide synthesizing innervation in primary achalasia | American Journal of Gastroenterology | Etiology | basic science research | 1999 | 80 |
| 143 | Wehrmann T. | Pneumatic dilation in achalasia with a low-compliance balloon: results of a 5-year prospective evaluation | Gastrointestinal Endoscopy | Treatment | clinical research | 1995 | 78 |
| 144 | Bonatti H. | Long-term results of laparoscopic Heller myotomy with partial fundoplication for the treatment of achalasia | American Journal of Surgery | Treatment | clinical research | 2005 | 78 |
| 145 | Patti M.G. | Comparison of Thoracoscopic and Laparoscopic Heller Myotomy for Achalasia | Journal of Gastrointestinal Surgery | Treatment | clinical research | 1998 | 78 |
| 146 | Metman E.-H. | Risk factors for immediate complications after progressive pneumatic dilation for achalasia | American Journal of Gastroenterology | Other | clinical research | 1999 | 77 |
| 147 | Gordon J.M. | Prospective study of esophageal botulinum toxin injection in high-risk achalasia patients | American Journal of Gastroenterology | Treatment | clinical research | 1997 | 77 |
| 148 | Verlaan T. | Effect of peroral endoscopic myotomy on esophagogastric junction physiology in patients with achalasia | Gastrointestinal Endoscopy | Treatment | clinical research | 2013 | 77 |
| 149 | Lyass S. | Current status of an antireflux procedure in laparoscopic Heller myotomy | Surgical Endoscopy and Other Interventional Techniques | Treatment | clinical research | 2003 | 77 |
| 150 | Podas T. | Achalasia: A critical review of epidemiological studies | American Journal of Gastroenterology | Other | review | 1998 | 77 |
| 151 | Portale, G | Long-term outcome of laparoscopic Heller-Dor surgery for esophageal achalasia: Possible detrimental role of previous endoscopic treatment | JOURNAL OF GASTROINTESTINAL SURGERY | Treatment | clinical research | 2005 | 76 |
| 152 | Patti M.G. | Laparoscopic Heller myotomy relieves dysphagia in achalasia when the esophagus is dilated | Surgical Endoscopy | Treatment | clinical research | 1999 | 76 |
| 153 | Facco M. | T cells in the myenteric plexus of achalasia patients show a skewed TCR repertoire and react to HSV-1 antigens | American Journal of Gastroenterology | Etiology | basic science research | 2008 | 75 |
| 154 | Raymond L. | Inflammatory aetiology of primary oesophageal achalasia: An immunohistochemical and ultrastructural study of Auerbach's plexus | Histopathology | Etiology | basic science research | 1999 | 75 |
| 155 | Eckardt A.J. | Current clinical approach to achalasia | World Journal of Gastroenterology | Treatment | clinical research | 2009 | 75 |
| 156 | Boeckxstaens G.E. | Achalasia: Virus-induced euthanasia of neurons? | American Journal of Gastroenterology | Etiology | basic science research | 2008 | 75 |
| 157 | Crookes P.F. | Gastroesophageal reflux in achalasia: When is reflux really reflux? | Digestive Diseases and Sciences | Diagnosis | clinical research | 1997 | 74 |
| 158 | Miller D.L. | Esophageal resection for recurrent achalasia | The Annals of Thoracic Surgery | Treatment | clinical research | 1995 | 74 |
| 159 | Birgisson S. | Achalasia: What’s new in diagnosis and treatment? | Digestive Diseases | All | review | 1997 | 74 |
| 160 | Hoffman B.J. | Treatment of achalasia by injection of botulinum toxin under endoscopic ultrasound guidance | Gastrointestinal Endoscopy | Treatment | clinical research | 1997 | 73 |
| 161 | Eckardt V.F. | Risk factors for diagnostic delay in achalasia | Digestive Diseases and Sciences | Other | clinical research | 1997 | 73 |
| 162 | Repici A. | GERD after per-oral endoscopic myotomy as compared with Heller's myotomy with fundoplication: a systematic review with meta-analysis | Gastrointestinal Endoscopy | Treatment | review | 2018 | 73 |
| 163 | Teitelbaum E.N. | Esophagogastric junction distensibility measurements during Heller myotomy and POEM for achalasia predict postoperative symptomatic outcomes | Surgical Endoscopy | Treatment | clinical research | 2015 | 73 |
| 164 | Kilic A. | Long-term outcomes of laparoscopic Heller myotomy for achalasia | Surgery | Treatment | clinical research | 2009 | 72 |
| 165 | Rosati R. | Laparoscopic approach to esophageal achalasia | American Journal of Surgery | Treatment | clinical research | 1995 | 72 |
| 166 | Gockel I. | Heller Myotomy for Failed Pneumatic Dilation in Achalasia: How Effective Is It? | Annals of Surgery | Treatment | clinical research | 2004 | 72 |
| 167 | Kumbhari V. | Gastroesophageal reflux after peroral endoscopic myotomy: A multicenter case-control study | Endoscopy | Treatment | clinical research | 2017 | 72 |
| 168 | Neubrand M. | Long-term results and prognostic factors in the treatment of achalasia with botulinum toxin | Endoscopy | Treatment | clinical research | 2002 | 71 |
| 169 | Leyden J.E. | Endoscopic pneumatic dilation versus botulinum toxin injection in the management of primary achalasia | Cochrane Database of Systematic Reviews | Treatment | clinical research | 2006 | 71 |
| 170 | Guardino J.M. | Pneumatic dilation for the treatment of achalasia in untreated patients and patients with failed Heller myotomy | Journal of Clinical Gastroenterology | Treatment | clinical research | 2004 | 70 |
| 171 | Minami H. | Peroral endoscopic myotomy for esophageal achalasia: Clinical impact of 28 cases | Digestive Endoscopy | Treatment | clinical research | 2014 | 70 |
| 172 | Bansal R. | Intrasphincteric botulinum toxin versus pneumatic balloon dilation for treatment of primary achalasia | Journal of Clinical Gastroenterology | Treatment | clinical research | 2003 | 70 |
| 173 | Patti M.G. | Importance of Preoperative and Postoperative pH Monitoring in Patients with Esophageal Achalasia | Journal of Gastrointestinal Surgery | Treatment | clinical research | 1997 | 70 |
| 174 | Eleftheriadis N. | Training in peroral endoscopic myotomy (POEM) for esophageal achalasia | Therapeutics and Clinical Risk Management | Treatment | clinical research | 2012 | 69 |
| 175 | O'Connor J.B. | The cost-effectiveness of treatment strategies for achalasia | Digestive Diseases and Sciences | Treatment | clinical research | 2002 | 69 |
| 176 | Patti M.G. | Spectrum of esophageal motility disorders: Implications for diagnosis and treatment | Archives of Surgery | All | clinical research | 2005 | 69 |
| 177 | Borotto E. | Risk factors of oesophageal perforation during pneumatic dilatation for achalasia | Gut | Other | clinical research | 1996 | 69 |
| 178 | Chen W.-F. | Long-term outcomes of peroral endoscopic myotomy for achalasia in pediatric patients: A prospective, single-center study | Gastrointestinal Endoscopy | Treatment | clinical research | 2015 | 69 |
| 179 | Wewalka F.W., Clodi P.H., Haidinger D. | Endoscopic clipping of esophageal perforation after pneumatic dilation for achalasia | Endoscopy | Treatment | clinical research | 1995 | 69 |
| 180 | Haito-Chavez Y. | Comprehensive Analysis of Adverse Events Associated with per Oral Endoscopic Myotomy in 1826 Patients: An International Multicenter Study | American Journal of Gastroenterology | Treatment | clinical research | 2017 | 69 |
| 181 | Verne G.N. | Association of HLA-DR and -DQ alleles with idiopathic achalasia | Gastroenterology | Etiology | basic science research | 1999 | 69 |
| 182 | Novais P.A. | 24-h pH monitoring patterns and clinical response after achalasia treatment with pneumatic dilation or laparoscopic Heller myotomy | Alimentary Pharmacology and Therapeutics | All | clinical research | 2010 | 69 |
| 183 | Vigneswaran Y. | Peroral Endoscopic Myotomy (POEM): Feasible as Reoperation Following Heller Myotomy | Journal of Gastrointestinal Surgery | Treatment | clinical research | 2014 | 68 |
| 184 | Hurwitz M. | Evaluation of the use of botulinum toxin in children with achalasia | Journal of Pediatric Gastroenterology and Nutrition | Treatment | clinical research | 2000 | 68 |
| 185 | Kraichely R.E. | Achalasia: Physiology and etiopathogenesis | Diseases of the Esophagus | All | review | 2006 | 68 |
| 186 | Ghoshal U.C. | Randomized controlled trial of intrasphincteric botulinum toxin A injection versus balloon dilatation in treatment of achalasia cardia | Diseases of the Esophagus | Treatment | clinical research | 2001 | 67 |
| 187 | Ponce J. | Individual prediction of response to pneumatic dilation in patients with achalasia | Digestive Diseases and Sciences | Treatment | clinical research | 1996 | 67 |
| 188 | Abir F. | Surgical treatment of achalasia: Current status and controversies | Digestive Surgery | Treatment | review | 2004 | 66 |
| 189 | Marano L. | Surgery or peroral esophageal myotomy for Achalasia: A systematic review and meta-analysis | Medicine (United States) | Treatment | review | 2016 | 66 |
| 190 | Virgilio C. | Endoscopic treatment of postoperative colonic strictures using an achalasia dilator: Short-term and long-term results | Endoscopy | Treatment | clinical research | 1995 | 66 |
| 191 | Patel K. | Peroral endoscopic myotomy for the treatment of esophageal achalasia: systematic review and pooled analysis | Diseases of the Esophagus | Treatment | review | 2016 | 65 |
| 192 | Storch W.B. | Autoantibodies to Auerbach's plexus in achalasia. | Cellular and molecular biology (Noisy-le-Grand, France) | Etiology | basic science research | 1995 | 65 |
| 193 | Birgisson S. | Achalasia is not associated with measles or known herpes and human papilloma viruses | Digestive Diseases and Sciences | Etiology | clinical research | 1997 | 65 |
| 194 | Teitelbaum, Ezra N. | Comparison of esophagogastric junction distensibility changes during POEM and Heller myotomy using intraoperative FLIP | SURGICAL ENDOSCOPY AND OTHER INTERVENTIONAL TECHNIQUES | Treatment | clinical research | 2013 | 64 |
| 195 | Richter J.E. | Update on the management of achalasia: Balloons, surgery and drugs | Expert Review of Gastroenterology and Hepatology | Treatment | review | 2008 | 64 |
| 196 | Hungness E.S. | Per-oral endoscopic myotomy (POEM) after the learning curve: Durable long-term results with a low complication rate | Annals of Surgery | Treatment | clinical research | 2016 | 64 |
| 197 | Rieder E. | Intraoperative assessment of esophagogastric junction distensibility during per oral endoscopic myotomy (POEM) for esophageal motility disorders | Surgical Endoscopy | Treatment | clinical research | 2013 | 64 |
| 198 | Rosati R. | Evaluating results of laparoscopic surgery for esophageal achalasia | Surgical Endoscopy | Treatment | clinical research | 1998 | 64 |
| 199 | Peters J.H. | Esophageal Resection With Colon Interposition for End-Stage Achalasia | Archives of Surgery | Treatment | clinical research | 1995 | 64 |
| 200 | Lake J.M. | Review article: The management of achalasia - A comparison of different treatment modalities | Alimentary Pharmacology and Therapeutics | Treatment | review | 2006 | 63 |
| 201 | Ngamruengphong S. | Efficacy and Safety of Peroral Endoscopic Myotomy for Treatment of Achalasia After Failed Heller Myotomy | Clinical Gastroenterology and Hepatology | Treatment | clinical research | 2017 | 63 |
| 202 | Morino M. | Preoperative pneumatic dilatation represents a risk factor for laparoscopic Heller myotomy | Surgical Endoscopy | Treatment | clinical research | 1997 | 62 |
| 203 | Annese V. | Intrasphincteric injection of botulinum toxin is effective in long-term treatment of esophageal achalasia | Muscle and Nerve | Treatment | clinical research | 1998 | 62 |
| 204 | Herve, Dominique | Loss of alpha 1 beta 1 Soluble Guanylate Cyclase, the Major Nitric Oxide Receptor, Leads to Moyamoya and Achalasia | AMERICAN JOURNAL OF HUMAN GENETICS | Etiology | basic science research | 2014 | 61 |
| 205 | Katzka D.A. | Review article: An analysis of the efficacy, perforation rates and methods used in pneumatic dilation for achalasia | Alimentary Pharmacology and Therapeutics | Treatment | review | 2011 | 61 |
| 206 | Hu J.-W. | Peroral endoscopic myotomy for advanced achalasia with sigmoid-shaped esophagus: long-term outcomes from a prospective, single-center study | Surgical Endoscopy | Treatment | clinical research | 2015 | 61 |
| 207 | Panaccione R. | Intrasphincteric botulinum toxin versus pneumatic dilatation for achalasia: A cost minimization analysis | Gastrointestinal Endoscopy | Treatment | clinical research | 1999 | 61 |
| 208 | Persson J. | Treatment of achalasia with laparoscopic myotomy or pneumatic dilatation: Long-term results of a prospective, randomized study | World Journal of Surgery | Treatment | clinical research | 2015 | 60 |
| 209 | Zendehdel K. | Risk of esophageal adenocarcinoma in achalasia patients, a retrospective cohort study in Sweden | American Journal of Gastroenterology | Other | clinical research | 2011 | 60 |
| 210 | Arain M.A. | Preoperative lower esophageal sphincter pressure affects outcome of laparoscopic esophageal myotomy for achalasia | Journal of Gastrointestinal Surgery | Treatment | clinical research | 2004 | 60 |
| 211 | Mikaeli J. | Pneumatic balloon dilatation in achalasia: A prospective comparison of safety and efficacy with different balloon diameters | Alimentary Pharmacology and Therapeutics | Treatment | clinical research | 2004 | 60 |
| 212 | Bechara R. | Per-oral endoscopic myotomy, 1000 cases later: pearls, pitfalls, and practical considerations | Gastrointestinal Endoscopy | Treatment | clinical research | 2016 | 60 |
| 213 | Orenstein S.B. | Peroral endoscopic myotomy (POEM) leads to similar results in patients with and without prior endoscopic or surgical therapy | Surgical Endoscopy | Treatment | clinical research | 2015 | 60 |
| 214 | Anselmino M. | One-year follow-up after laparoscopic Heller-Dor operation for esophageal achalasia | Surgical Endoscopy | Treatment | clinical research | 1997 | 60 |
| 215 | Annese V. | Comparison of two different formulations of botulinum toxin A for the treatment of oesophageal achalasia | Alimentary Pharmacology and Therapeutics | Treatment | clinical research | 1999 | 60 |
| 216 | Teitelbaum E.N. | Analysis of a Learning Curve and Predictors of Intraoperative Difficulty for Peroral Esophageal Myotomy (POEM) | Journal of Gastrointestinal Surgery | Treatment | clinical research | 2014 | 60 |
| 217 | Gockel H.R. | Achalasia: Will genetic studies provide insights? | Human Genetics | Etiology | review | 2010 | 60 |
| 218 | Lehman M.B. | Squamous mucosal alterations in esophagectomy specimens from patients with end-stage achalasia | American Journal of Surgical Pathology | Other | basic science research | 2001 | 59 |
| 219 | Gockel I. | Reduction of interstitial cells of Cajal (ICC) associated with neuronal nitric oxide synthase (n-NOS) in patients with achalasia | American Journal of Gastroenterology | Etiology | basic science research | 2008 | 59 |
| 220 | Roman S. | High-resolution manometry improves the diagnosis of esophageal motility disorders in patients with dysphagia: A randomized multicenter study | American Journal of Gastroenterology | Diagnosis | clinical research | 2016 | 59 |
| 221 | Urbach D.R. | A measure of disease-specific health-related quality of life for achalasia | American Journal of Gastroenterology | Other | clinical research | 2005 | 59 |
| 222 | Rosemurgy, A | Laparoscopic Heller myotomy provides durable relief from achalasia and salvages failures after Botox or dilation | ANNALS OF SURGERY | Treatment | clinical research | 2005 | 58 |
| 223 | Carlson D.A. | The Functional Lumen Imaging Probe Detects Esophageal Contractility Not Observed with Manometry in Patients with Achalasia | Gastroenterology | Diagnosis | clinical research | 2015 | 58 |
| 224 | Khan A.A. | Pneumatic balloon dilation in achalasia: A prospective comparison of balloon distention time | American Journal of Gastroenterology | Treatment | clinical research | 1998 | 58 |
| 225 | Kadakia S.C. | Pneumatic balloon dilation for esophageal achalasia | Gastrointestinal Endoscopy Clinics of North America | Treatment | review | 2001 | 58 |
| 226 | Finley R.J. | Laparoscopic Heller myotomy improves esophageal emptying and the symptoms of achalasia | Archives of Surgery | Treatment | clinical research | 2001 | 58 |
| 227 | Anselmino M. | Heller myotomy is superior to dilatation for the treatment of early achalasia | Archives of Surgery | Treatment | clinical research | 1997 | 58 |
| 228 | Alderliesten J. | Predictors for outcome of failure of balloon dilatation in patients with achalasia | Gut | Treatment | clinical research | 2011 | 57 |
| 229 | Ben-Meir A. | Quality of life before and after laparoscopic Heller myotomy for achalasia | American Journal of Surgery | Treatment | clinical research | 2001 | 56 |
| 230 | Gideon R.M. | Prospective randomized comparison of pneumatic dilatation technique in patients with idiopathic achalasia | Digestive Diseases and Sciences | Treatment | clinical research | 1999 | 56 |
| 231 | Hoogerwerf W.A. | Pharmacologic therapy in treating achalasia | Gastrointestinal Endoscopy Clinics of North America | Treatment | review | 2001 | 56 |
| 232 | Lee B.H. | Peroral endoscopic myotomy for treatment of achalasia: Initial results of a Korean study | Clinical Endoscopy | Treatment | clinical research | 2013 | 56 |
| 233 | De Caluwé D. | Internal anal sphincter achalasia: Outcome after internal sphincter myectomy | Journal of Pediatric Surgery | Treatment | clinical research | 2001 | 56 |
| 234 | Woodfield C.A. | Diagnosis of primary versus secondary achalasia: Reassessment of clinical and radiographic criteria | American Journal of Roentgenology | Diagnosis | clinical research | 2000 | 56 |
| 235 | Khelif K. | Achalasia of the cardia in Allgrove's (triple A) syndrome: Histopathologic study of 10 cases | American Journal of Surgical Pathology | Other | clinical research | 2003 | 56 |
| 236 | Rosemurgy A.S. | A Single Institution's Experience with More than 500 Laparoscopic Heller Myotomies for Achalasia | Journal of the American College of Surgeons | Treatment | clinical research | 2010 | 56 |
| 237 | Kostic S.V., Rice T.W., Baker M.E., DeCamp M.M. | Timed barium esophagogram: A simple physiologic assessment for achalasia | Journal of Thoracic and Cardiovascular Surgery | Diagnosis | clinical research | 2000 | 55 |
| 238 | Barbieri L.A. | Systematic review and meta-analysis: Efficacy and safety of POEM for achalasia | United European Gastroenterology Journal | Treatment | review | 2015 | 55 |
| 239 | Beckingham I.J. | Laparoscopic cardiomyotomy for achalasia after failed balloon dilatation | Surgical Endoscopy | Treatment | clinical research | 1999 | 55 |
| 240 | Farrokhi F. | Idiopathic (primary) achalasia | Orphanet Journal of Rare Diseases | All | review | 2007 | 55 |
| 241 | Shaligram A. | How does the robot affect outcomes? A retrospective review of open, laparoscopic, and robotic Heller myotomy for achalasia | Surgical Endoscopy | Treatment | review | 2012 | 55 |
| 242 | Gockel I. | Common variants in the HLA-DQ region confer susceptibility to idiopathic achalasia | Nature Genetics | Etiology | basic science research | 2014 | 55 |
| 243 | Ghoshal U.C. | Pathogenesis of achalasia cardia | World Journal of Gastroenterology | Pathogenesis | review | 2012 | 54 |
| 244 | Eckardt V.F. | Complications and their impact after pneumatic dilation for achalasia: Prospective long-term follow-up study | Gastrointestinal Endoscopy | Treatment | clinical research | 1997 | 54 |
| 245 | Chapman J.R. | Achalasia Treatment: Improved Outcome of Laparoscopic Myotomy with Operative Manometry | Archives of Surgery | Treatment | clinical research | 2004 | 54 |
| 246 | Lee C.W. | Outcomes of treatment of childhood achalasia | Journal of Pediatric Surgery | Treatment | clinical research | 2010 | 53 |
| 247 | D'Onofrio V. | Long-term follow-up of achalasia patients treated with botulinum toxin | Digestive and Liver Disease | Treatment | clinical research | 2002 | 53 |
| 248 | Iqbal A. | Laparoscopic re-operation for failed Heller myotomy | Diseases of the Esophagus | Treatment | clinical research | 2006 | 53 |
| 249 | Werner Y.B. | Endoscopic or surgical myotomy in patients with idiopathic Achalasia | New England Journal of Medicine | Treatment | clinical research | 2019 | 53 |
| 250 | Walzer N. | Achalasia | Gastroenterology Clinics of North America | All | review | 2008 | 53 |
| 251 | Frantzides, CT | Minimally invasive surgery for achalasia: A 10-year experience | JOURNAL OF GASTROINTESTINAL SURGERY | Treatment | clinical research | 2004 | 52 |
| 252 | Teitelbaum E.N. | Peroral esophageal myotomy (POEM) and laparoscopic Heller myotomy produce a similar short-term anatomic and functional effect | Surgery (United States) | Treatment | clinical research | 2013 | 52 |
| 253 | Schoenberg M.B. | Laparoscopic Heller Myotomy versus Endoscopic Balloon Dilatation for the treatment of achalasia: A network meta-analysis | Annals of Surgery | Treatment | review | 2013 | 52 |
| 254 | Familiari P. | Gastroesophageal reflux disease after peroral endoscopic myotomy: Analysis of clinical, procedural and functional factors, associated with gastroesophageal reflux disease and esophagitis | Digestive Endoscopy | Other | clinical research | 2016 | 52 |
| 255 | Rohof W.O. | Esophageal Stasis on a timed barium esophagogram predicts recurrent symptoms in patients with long-standing achalasia | American Journal of Gastroenterology | Diagnosis | clinical research | 2013 | 52 |
| 256 | Naef M. | Esophageal dysmotility disorders after laparoscopic gastric banding-an underestimated complication | Annals of Surgery | Treatment | clinical research | 2011 | 52 |
| 257 | Ponds F.A. | Effect of Peroral Endoscopic Myotomy vs Pneumatic Dilation on Symptom Severity and Treatment Outcomes among Treatment-Naive Patients with Achalasia: A Randomized Clinical Trial | JAMA - Journal of the American Medical Association | Treatment | clinical research | 2019 | 52 |
| 258 | Urbach D.R. | A Decision Analysis of the Optimal Initial Approach to Achalasia: Laparoscopic Heller Myotomy with Partial Fundoplication, Thoracoscopic Heller Myotomy, Pneumatic Dilatation, or Botulinum Toxin Injection | Journal of Gastrointestinal Surgery | Treatment | clinical research | 2001 | 52 |
| 259 | Chan K.C. | Short-term and long-term results of endoscopic balloon dilation for achalasia: 12 years' experience | Endoscopy | Treatment | clinical research | 2004 | 51 |
| 260 | Cai M.-Y. | Peroral endoscopic myotomy for idiopathic achalasia: Randomized comparison of water-jet assisted versus conventional dissection technique | Surgical Endoscopy | Treatment | clinical research | 2014 | 51 |
| 261 | Snyder C.W. | Multiple preoperative endoscopic interventions are associated with worse outcomes after laparoscopic Heller myotomy for achalasia | Journal of Gastrointestinal Surgery | Treatment | clinical research | 2009 | 51 |
| 262 | Hervé D. | Loss of α1β1 soluble guanylate cyclase, the major nitric oxide receptor, leads to moyamoya and achalasia | American Journal of Human Genetics | Etiology | basic science research | 2014 | 51 |
| 263 | Katsinelos P. | Long-term results of pneumatic dilation for achalasia: A 15 years' experience | World Journal of Gastroenterology | Treatment | clinical research | 2005 | 51 |
| 264 | Ackroyd R. | Laparoscopic cardiomyotomy and anterior partial fundoplication for achalasia | Surgical Endoscopy | Treatment | clinical research | 2001 | 51 |
| 265 | Ciamarra P. | Internal anal sphincter achalasia in children: Clinical characteristics and treatment with Clostridium botulinum toxin | Journal of Pediatric Gastroenterology and Nutrition | All | clinical research | 2003 | 51 |
| 266 | Patti M.G. | Fundoplication After Laparoscopic Heller Myotomy for Esophageal Achalasia: What Type? | Journal of Gastrointestinal Surgery | Treatment | review | 2010 | 51 |
| 267 | Douard R. | Functional results after laparoscopic Heller myotomy for achalasia: A comparative study to open surgery | Surgery | Treatment | clinical research | 2004 | 51 |
| 268 | Marlais M. | UK incidence of achalasia: An 11-year national epidemiological study | Archives of Disease in Childhood | Other | clinical research | 2011 | 50 |
| 269 | Bruley Des Varannes S. | Serum from achalasia patients alters neurochemical coding in the myenteric plexus and nitric oxide mediated motor response in normal human fundus | Gut | Etiology | basic science research | 2006 | 50 |
| 270 | Zhao J.-G. | Long-term safety and outcome of a temporary self-expanding metallic stent for achalasia: A prospective study with a 13-year single-center experience | European Radiology | Treatment | clinical research | 2009 | 50 |
| 271 | Ali A. | Laparoscopic myotomy: Technique and efficacy in treating achalasia | Gastrointestinal Endoscopy Clinics of North America | Treatment | review | 2001 | 50 |
| 272 | Rothenberg S.S. | Evaluation of minimally invasive approaches to achalasia in children | Journal of Pediatric Surgery | Treatment | clinical research | 2001 | 50 |
| 273 | Teitelbaum E.N. | Clinical outcomes five years after POEM for treatment of primary esophageal motility disorders | Surgical Endoscopy | Treatment | clinical research | 2018 | 50 |
| 274 | Niwamoto H. | Are human herpes viruses or measles virus associated with esophageal achalasia? | Digestive Diseases and Sciences | Etiology | clinical research | 1995 | 50 |
| 275 | Blam M.E. | Achalasia: A disease of varied and subtle symptoms that do not correlate with radiographic findings | American Journal of Gastroenterology | Other | review | 2002 | 50 |
| 276 | Zaheed Hussain S. | A review of achalasia in 33 children | Digestive Diseases and Sciences | All | review | 2002 | 50 |
| 277 | Eaker E.Y. | Untoward effects of esophageal botulinum toxin injection in the treatment of achalasia | Digestive Diseases and Sciences | Treatment | clinical research | 1997 | 49 |
| 278 | Jeansonne L.O. | Ten-year follow-up of laparoscopic Heller myotomy for achalasia shows durability | Surgical Endoscopy and Other Interventional Techniques | Treatment | clinical research | 2007 | 49 |
| 279 | Bassotti G. | Pharmacological options in achalasia | Alimentary Pharmacology and Therapeutics | Treatment | review | 1999 | 49 |
| 280 | Torbey C.F. | Long-term outcome of achalasia treatment: The need for closer follow-up | Journal of Clinical Gastroenterology | Treatment | clinical research | 1999 | 49 |
| 281 | Eckardt V.F. | Life expectancy, complications, and causes of death in patients with achalasia: Results of a 33-year follow-up investigation | European Journal of Gastroenterology and Hepatology | Other | clinical research | 2008 | 49 |
| 282 | Pechlivanides G. | Laparoscopic Heller cardiomyotomy and Dor fundoplication for esophageal achalasia: Possible factors predicting outcome | Archives of Surgery | Treatment | clinical research | 2001 | 49 |
| 283 | Goin J.C. | Functional implications of circulating muscarinic cholinergic receptor autoantibodies in chagasic patients with achalasia | Gastroenterology | Etiology | basic science research | 1999 | 49 |
| 284 | Kahrilas P.J. | Clinical Practice Update: The Use of Per-Oral Endoscopic Myotomy in Achalasia: Expert Review and Best Practice Advice From the AGA Institute | Gastroenterology | Treatment | guideline and consensus | 2017 | 49 |
| 285 | Schiano T.D. | Use of high-resolution endoscopic ultrasonography to assess esophageal wall damage after pneumatic dilation and botulinum toxin injection to treat achalasia | Gastrointestinal Endoscopy | Treatment | clinical research | 1996 | 48 |
| 286 | Gockel I. | Spectrum of histopathologic findings in patients with achalasia reflects different etiologies | Journal of Gastroenterology and Hepatology (Australia) | Etiology | basic science research | 2006 | 48 |
| 287 | De Palma G.D. | Self-expanding metal stents for endoscopic treatment of esophageal achalasia unresponsive to conventional treatments. Long-term results in eight patients | Endoscopy | Treatment | clinical research | 2001 | 48 |
| 288 | Deb S. | Laparoscopic esophageal myotomy for achalasia: Factors affecting functional results | Annals of Thoracic Surgery | Treatment | clinical research | 2005 | 48 |
| 289 | Chen Z. | Laparoscopic cardiomyotomy for achalasia: Clinical outcomes beyond 5 years | Journal of Gastrointestinal Surgery | Treatment | clinical research | 2010 | 48 |
| 290 | Enestvedt B.K. | Epidemiology and practice patterns of achalasia in a large multi-centre database | Alimentary Pharmacology and Therapeutics | Other | clinical research | 2011 | 48 |
| 291 | Prakash C. | Botulinum toxin injections for achalasia symptoms can approximate the short term efficacy of a single pneumatic dilation: A survival analysis approach | American Journal of Gastroenterology | Treatment | clinical research | 1999 | 48 |
| 292 | Zárate N. | Achalasia and Down's syndrome: Coincidential association or something else? | American Journal of Gastroenterology | Other | clinical research | 1999 | 48 |
| 293 | Martinez-Gonzalez, Daniel | ACHALASIA AND MYCOBACTERIUM GOODII PULMONARY INFECTION | PEDIATRIC INFECTIOUS DISEASE JOURNAL | Other | clinical research | 2011 | 47 |
| 294 | Wang Y.R. | Trends of Heller myotomy hospitalizations for achalasia in the United States, 1993-2005: Effect of surgery volume on perioperative outcomes | American Journal of Gastroenterology | Treatment | clinical research | 2008 | 47 |
| 295 | Wang P.C. | The outcome of laparoscopic Heller myotomy without antireflux procedure in patients with achalasia | American Surgeon | Treatment | clinical research | 1998 | 47 |
| 296 | Familiari P. | Peroral endoscopic myotomy for the treatment of achalasia in children | Journal of Pediatric Gastroenterology and Nutrition | Treatment | clinical research | 2013 | 47 |
| 297 | Dobrucali A. | Long-term results of graded pneumatic dilatation under endoscopic guidance in patients with primary esophageal achalasia | World Journal of Gastroenterology | Treatment | clinical research | 2004 | 47 |
| 298 | Paterson W.G. | Etiology and pathogenesis of achalasia | Gastrointestinal Endoscopy Clinics of North America | All | review | 2001 | 47 |
| 299 | Chan S.M. | Comparison of early outcomes and quality of life after laparoscopic Heller's cardiomyotomy to peroral endoscopic myotomy for treatment of achalasia | Digestive Endoscopy | Treatment | clinical research | 2016 | 47 |
| 300 | Maselli, R. | Peroral endoscopic myotomy (POEM) in a 3-year-old girl with severe growth retardation, achalasia, and Down syndrome | ENDOSCOPY | Treatment | clinical research | 2012 | 46 |
| 301 | Martínek J. | Treatment of patients with achalasia with botulinum toxin: A multicenter prospective cohort study | Diseases of the Esophagus | Treatment | clinical research | 2003 | 46 |
| 302 | Mattioli G. | Results of the laparoscopic Heller-Dor procedure for pediatric esophageal achalasia | Surgical Endoscopy and Other Interventional Techniques | Treatment | clinical research | 2003 | 46 |
| 303 | Lambroza A. | Pneumatic Dilation for Achalasia without Fluoroscopic Guidance: Safety and Efficacy | The American Journal of Gastroenterology | Treatment | clinical research | 1995 | 46 |
| 304 | Holzman M.D. | Laparoscopic surgical treatment of achalasia | American Journal of Surgery | Treatment | clinical research | 1997 | 46 |
| 305 | Galvani C. | Laparoscopic Heller myotomy for achalasia facilitated by robotic assistance | Surgical Endoscopy and Other Interventional Techniques | Treatment | clinical research | 2006 | 46 |
| 306 | Yamamura M.S. | Laparoscopic Heller myotomy and anterior fundoplication for achalasia results in a high degree of patient satisfaction | Archives of Surgery | Treatment | clinical research | 2000 | 46 |
| 307 | Andersson M. | Evaluation of the response to treatment in patients with idiopathic achalasia by the timed barium esophagogram: Results from a randomized clinical trial | Diseases of the Esophagus | Treatment | clinical research | 2009 | 46 |
| 308 | Stylopoulos N. | Development of achalasia secondary to laparoscopic nissen fundoplication | Journal of Gastrointestinal Surgery | Other | clinical research | 2002 | 46 |
| 309 | Dempsey D.T. | Comparison of outcomes following open and laparoscopic esophagomyotomy for achalasia | Surgical Endoscopy | Treatment | clinical research | 1999 | 46 |
| 310 | Herbella F.A.M. | Are idiopathic and chagasic achalasia two different diseases? | Digestive Diseases and Sciences | Other | review | 2004 | 46 |
| 311 | Hunter J.G. | Surgical management of achalasia | Surgical Clinics of North America | Treatment | review | 1997 | 45 |
| 312 | Mattioli S. | Surgery for Esophageal Achalasia. Long-term Results with Three Different Techniques | Hepato-Gastroenterology | Treatment | clinical research | 1996 | 45 |
| 313 | Crespin O.M. | Safety and efficacy of POEM for treatment of achalasia: a systematic review of the literature | Surgical Endoscopy | Treatment | review | 2017 | 45 |
| 314 | Dunaway P.M. | Risk and surveillance intervals for squamous cell carcinoma in achalasia | Gastrointestinal Endoscopy Clinics of North America | Other | review | 2001 | 45 |
| 315 | Gorecki P.J. | Redo laparoscopic surgery for achalasia: Is it feasible? | Surgical Endoscopy and Other Interventional Techniques | Treatment | clinical research | 2002 | 45 |
| 316 | Zarate N. | Intramuscular interstitial cells of Cajal associated with mast cells survive nitrergic nerves in achalasia | Neurogastroenterology and Motility | Etiology | basic science research | 2006 | 45 |
| 317 | Imperiale T.F. | Cost-minimization analysis of alternative treatment strategies for achalasia | American Journal of Gastroenterology | Treatment | review | 2000 | 45 |
| 318 | Khashab M.A. | Comprehensive analysis of efficacy and safety of peroral endoscopic myotomy performed by a gastroenterologist in the endoscopy unit: A single-center experience | Gastrointestinal Endoscopy | Treatment | clinical research | 2016 | 45 |
| 319 | Booy J.D. | The prevalence of autoimmune disease in patients with esophageal achalasia | Diseases of the Esophagus | Other | clinical research | 2012 | 44 |
| 320 | Burpee S.E. | Objective analysis of gastroesophageal reflux after laparoscopic Heller myotomy: An anti-reflux procedure is required | Surgical Endoscopy and Other Interventional Techniques | Treatment | clinical research | 2005 | 44 |
| 321 | Sodikoff J.B | Histopathologic patterns among achalasia subtypes | Neurogastroenterology and Motility | Other | basic science research | 2016 | 44 |
| 322 | Gennaro N. | Esophageal Achalasia in the Veneto Region: Epidemiology and Treatment | Journal of Gastrointestinal Surgery | All | clinical research | 2011 | 44 |
| 323 | Duranceau A. | End-stage achalasia | Diseases of the Esophagus | All | review | 2012 | 44 |
| 324 | Stavropoulos S.N. | Endoscopic submucosal myotomy for the treatment of achalasia (with video) | Gastrointestinal Endoscopy | Treatment | clinical research | 2010 | 44 |
| 325 | Loviscek L.F. | Early cancer in achalasia | Diseases of the Esophagus | Other | review | 1998 | 44 |
| 326 | Cheng Y.-S. | Selection and evaluation of three interventional procedures for achalasia based on long-term follow-up | World Journal of Gastroenterology | Treatment | clinical research | 2003 | 43 |
| 327 | Richards W.O. | Prevalence of gastroesophageal reflux after laparoscopic Heller myotomy | Surgical Endoscopy | Treatment | clinical research | 1999 | 43 |
| 328 | Pandolfino J.E. | Presentation, diagnosis, and management of achalasia | Clinical Gastroenterology and Hepatology | All | review | 2013 | 43 |
| 329 | Bechara R. | Peroral endoscopic myotomy: An evolving treatment for Achalasia | Nature Reviews Gastroenterology and Hepatology | Treatment | review | 2015 | 43 |
| 330 | Ramchandani M. | Peroral endoscopic myotomy for achalasia cardia: Treatment analysis and follow up of over 200 consecutive patients at a single center | Digestive Endoscopy | Treatment | clinical research | 2016 | 43 |
| 331 | Kraichely R.E. | Neural autoantibody profile of primary achalasia | Digestive Diseases and Sciences | Etiology | basic science research | 2010 | 43 |
| 332 | Castagliuolo I. | Esophageal achalasia: Is the herpes simplex virus really innocent? | Journal of Gastrointestinal Surgery | Etiology | basic science research | 2004 | 43 |
| 333 | Ling T.S. | Effectiveness of peroral endoscopic myotomy in the treatment of achalasia: A pilot trial in Chinese Han population with a minimum of one-year follow-up | Journal of Digestive Diseases | Treatment | clinical research | 2014 | 43 |
| 334 | Chuah S.-H. | 2011 update on esophageal achalasia | World Journal of Gastroenterology | All | review | 2012 | 43 |
| 335 | Ortiz A. | Very long-term objective evaluation of Heller myotomy plus posterior partial fundoplication in patients with achalasia of the cardia | Annals of Surgery | Treatment | clinical research | 2008 | 42 |
| 336 | Roman S. | Partial recovery of peristalsis after myotomy for achalasia: More the rule than the exception | JAMA Surgery | Other | clinical research | 2013 | 42 |
| 337 | Wills V.L. | Functional Outcome after Heller Myotomy and Fundoplication for Achalasia | Journal of Gastrointestinal Surgery | Treatment | clinical research | 2001 | 42 |
| 338 | Kessing B.F. | Erroneous diagnosis of gastroesophageal reflux disease in achalasia | Clinical Gastroenterology and Hepatology | Other | review | 2011 | 42 |
| 339 | Vanuytsel T. | Conservative Management of Esophageal Perforations During Pneumatic Dilation for Idiopathic Esophageal Achalasia | Clinical Gastroenterology and Hepatology | Treatment | clinical research | 2012 | 42 |
| 340 | Hirakawa H. | Absence of nadph-diaphorase activity in internal anal sphincter (Ias) achalasia | Journal of Pediatric Gastroenterology and Nutrition | Etiology | basic science research | 1995 | 42 |
| 341 | Nicodème F. | A Comparison of Symptom Severity and Bolus Retention With Chicago Classification Esophageal Pressure Topography Metrics in Patients With Achalasia | Clinical Gastroenterology and Hepatology | Diagnosis | clinical research | 2013 | 42 |
| 342 | Chapman, JR | Achalasia treatment - Improved outcome of laparoscopic myotomy with operative manometry | ARCHIVES OF SURGERY | Treatment | clinical research | 2004 | 41 |
| 343 | Annese V. | Perendoscopic injection of botulinum toxin is effective in achalasia after failure of myotomy or pneumatic dilation | Gastrointestinal Endoscopy | Treatment | clinical research | 1996 | 41 |
| 344 | Zaninotto G. | Long-term outcome and risk of oesophageal cancer after surgery for achalasia | British Journal of Surgery | Treatment | clinical research | 2008 | 41 |
| 345 | Hallal C. | Diagnosis, misdiagnosis, and associated diseases of achalasia in children and adolescents: A twelve-year single center experience | Pediatric Surgery International | All | clinical research | 2012 | 41 |
| 346 | Zárate N. | Achalasia treatment in the elderly: Is botulinum toxin injection the best option? | European Journal of Gastroenterology and Hepatology | Treatment | clinical research | 2002 | 41 |
| 347 | Birgisson S. | Achalasia in Iceland, 1952-2002: An epidemiologic study | Digestive Diseases and Sciences | Other | clinical research | 2007 | 41 |
| 348 | Woltman T.A. | Achalasia | Surgical Clinics of North America | All | review | 2005 | 41 |
| 349 | Caldaro, Tamara | Treatment of esophageal achalasia in children: Today and tomorrow | JOURNAL OF PEDIATRIC SURGERY | Treatment | clinical research | 2015 | 40 |
| 350 | Cheng Y.S. | Temporary self-expanding metallic stents for achalasia: a prospective study with a long-term follow-up. | World journal of gastroenterology : WJG | Treatment | clinical research | 2010 | 40 |
| 351 | Perrone J.M. | Results of laparoscopic Heller-Toupet operation for achalasia | Surgical Endoscopy and Other Interventional Techniques | Treatment | clinical research | 2004 | 40 |
| 352 | Zhang Y. | Per-Oral Endoscopic Myotomy Versus Laparoscopic Heller Myotomy for Achalasia | Medicine (United States) | Treatment | clinical research | 2016 | 40 |
| 353 | Di Simone M.P. | Onset timing of delayed complications and criteria of follow-up after operation for esophageal achalasia | Annals of Thoracic Surgery | Other | clinical research | 1996 | 40 |
| 354 | Andrews C.N. | Laparoscopic Heller's myotomy or botulinum toxin injection for management of esophageal achalasia: Patient choice and treatment outcomes | Surgical Endoscopy | Treatment | clinical research | 1999 | 40 |
| 355 | Sonnenberg A. | Hospitalization for achalasia in the United States 1997-2006 | Digestive Diseases and Sciences | Treatment | clinical research | 2009 | 40 |
| 356 | Banbury M.K. | Esophagectomy with gastric reconstruction for achalasia | Journal of Thoracic and Cardiovascular Surgery | Treatment | clinical research | 1999 | 40 |
| 357 | Chino O. | Clinicopathological studies of esophageal carcinoma in achalasia: Analyses of carcinogenesis using histological and immunohistochemical procedures | Anticancer Research | Other | basic science research | 2000 | 40 |
| 358 | Teitelbaum E.N. | An extended proximal esophageal myotomy is necessary to normalize EGJ distensibility during Heller myotomy for achalasia, but not POEM | Surgical Endoscopy | Treatment | clinical research | 2014 | 40 |
| 359 | Pastor A.C. | A single center 26-year experience with treatment of esophageal achalasia: is there an optimal method? | Journal of Pediatric Surgery | Treatment | clinical research | 2009 | 40 |
| 360 | Inoue, Haruhiro | Peroral endoscopic myotomy and fundoplication: a novel NOTES procedure | ENDOSCOPY | Treatment | clinical research | 2019 | 39 |
| 361 | Boztas G. | Pneumatic balloon dilatation in primary achalasia: The long-term follow-up results | Hepato-Gastroenterology | Treatment | clinical research | 2005 | 39 |
| 362 | Litle V.R. | Laparoscopic Heller Myotomy for Achalasia: A Review of the Controversies | Annals of Thoracic Surgery | Treatment | review | 2008 | 39 |
| 363 | Ngamruengphong S.. | Intraoperative measurement of esophagogastric junction cross-sectional area by impedance planimetry correlates with clinical outcomes of peroral endoscopic myotomy for achalasia: a multicenter study | Surgical Endoscopy | Treatment | clinical research | 2016 | 39 |
| 364 | Nguyen H.N. | Impedance characteristics of esophageal motor function in achalasia | Diseases of the Esophagus | Other | clinical research | 2004 | 39 |
| 365 | Parrilla P. | Factors Involved in the Return of Peristalsis in Patients with Achalasia of the Cardia after Heller's Myotomy | The American Journal of Gastroenterology | Other | clinical research | 1995 | 39 |
| 366 | Ponds F.A. | Esophagogastric junction distensibility identifies achalasia subgroup with manometrically normal esophagogastric junction relaxation | Neurogastroenterology and Motility | Diagnosis | clinical research | 2017 | 39 |
| 367 | Familiari P. | EndoFLIP system for the intraoperative evaluation of peroral endoscopic myotomy | United European Gastroenterology Journal | Treatment | clinical research | 2014 | 39 |
| 368 | Bonavina L. | Does previous endoscopic treatment affect the outcome of laparoscopic Heller myotomy? | Annales de Chirurgie | Treatment | clinical research | 2000 | 39 |
| 369 | Karnak I. | Achalasia in childhood: Surgical treatment and outcome | European Journal of Pediatric Surgery | Treatment | review | 2001 | 39 |
| 370 | Shoenut J.P. | A prospective assessment of gastroesophageal reflux before and after treatment of achalasia patients: Pneumatic dilation versus transthoracic limited myotomy | American Journal of Gastroenterology | Treatment | clinical research | 1997 | 39 |
| 371 | Bravi I. | A pneumatic dilation strategy in achalasia: Prospective outcome and effects on oesophageal motor function in the long term | Alimentary Pharmacology and Therapeutics | Treatment | clinical research | 2010 | 39 |
| 372 | Chen X. | Two-year follow-up for 45 patients with achalasia who underwent peroral endoscopic myotomy | European Journal of Cardio-thoracic Surgery | Treatment | clinical research | 2015 | 38 |
| 373 | Herbella F.A.M. | Treatment of achalasia: Lessons learned with Chagas'disease | Diseases of the Esophagus | Treatment | review | 2008 | 38 |
| 374 | Gockel I. | The value of scoring achalasia: A comparison of current systems and the impact on treatment - The surgeons viewpoint | American Surgeon | Treatment | clinical research | 2007 | 38 |
| 375 | Simić A.P. | Significance of limited hiatal dissection in surgery for achalasia | Journal of Gastrointestinal Surgery | Treatment | clinical research | 2010 | 38 |
| 376 | Muehldorfer S.M. | High- and low-compliance balloon dilators in patients with achalasia: A randomized prospective comparative trial | Gastrointestinal Endoscopy | Treatment | clinical research | 1996 | 38 |
| 377 | Donahue P.E. | Floppy Dor fundoplication after esophagocardiomyotomy for achalasia | Surgery | Treatment | clinical research | 2002 | 38 |
| 378 | Ling T. | Effect of peroral endoscopic myotomy in achalasia patients with failure of prior pneumatic dilation: A prospective case-control study | Journal of Gastroenterology and Hepatology (Australia) | Treatment | clinical research | 2014 | 38 |
| 379 | De La Concha E.G. | Contribution of HLA class II genes to susceptibility in achalasia | Tissue Antigens | Etiology | basic science research | 1998 | 38 |
| 380 | Zaninotto G. | Botulinum toxin injection vs laparoscopic myotomy for the treatment of esophageal achalasia: Economic analysis of a randomized trial | Surgical Endoscopy and Other Interventional Techniques | Treatment | clinical research | 2004 | 38 |
| 381 | Boeckxstaens G.E.E. | Achalasia | Best Practice and Research in Clinical Gastroenterology | All | review | 2007 | 38 |
| 382 | Patti, MG | Laparoscopic Heller myotomy and Dor fundoplication for esophageal achalasia in children | JOURNAL OF PEDIATRIC SURGERY | Treatment | clinical research | 2001 | 37 |
| 383 | Bassotti, G | Review article: pharmacological options in achalasia | ALIMENTARY PHARMACOLOGY & THERAPEUTICS | Treatment | review | 1999 | 37 |
| 384 | Camacho-Lobato L. | Vigorous achalasia: Original description requires minor change | Journal of Clinical Gastroenterology | Other | clinical research | 2001 | 37 |
| 385 | Katzka D.A. | Use of botulinum toxin as a diagnostic/therapeutic trial to help clarify an indication for definitive therapy in patients with achalasia | American Journal of Gastroenterology | Treatment | clinical research | 1999 | 37 |
| 386 | Ates F. | The pathogenesis and management of Achalasia: Current status and future directions | Gut and Liver | pathogenesis | review | 2015 | 37 |
| 387 | Triadafilopoulos G. | The Kagoshima consensus on esophageal achalasia | Diseases of the Esophagus | Treatment | clinical research | 2012 | 37 |
| 388 | Shiwaku H. | Peroral endoscopic myotomy for esophageal achalasia: outcomes of the first over 100 patients with short-term follow-up | Surgical Endoscopy | Treatment | clinical research | 2016 | 37 |
| 389 | Zaninotto G. | Minimally invasive surgery for esophageal achalasia | Journal of Laparoendoscopic and Advanced Surgical Techniques - Part A | Treatment | review | 2001 | 37 |
| 390 | Liu J.-F. | Long-term outcome of esophageal myotomy for achalasia | World Journal of Gastroenterology | Treatment | clinical research | 2004 | 37 |
| 391 | Chen L.-Q. | Long-term effects of myotomy and partial fundoplication for esophageal achalasia | Diseases of the Esophagus | Treatment | clinical research | 2002 | 37 |
| 392 | Cowgill S.M. | Laparoscopic Heller myotomy for achalasia: Results after 10 years | Surgical Endoscopy | Treatment | clinical research | 2009 | 37 |
| 393 | Rollan A. | Endoscopic intrasphincteric injection of botulinurn toxin for the treatment of achalasia | Journal of Clinical Gastroenterology | Treatment | clinical research | 1995 | 37 |
| 394 | Li Q.-L. | Early diagnosis and management of delayed bleeding in the submucosal tunnel after peroral endoscopic myotomy for achalasia (with video) | Gastrointestinal Endoscopy | Other | clinical research | 2013 | 37 |
| 395 | Williams V.A. | Achalasia of the Esophagus: A Surgical Disease | Journal of the American College of Surgeons | All | review | 2009 | 37 |
| 396 | Almogy G. | Achalasia in the Context of Morbid Obesity: A Rare but Important Association | Obesity Surgery | Treatment | clinical research | 2003 | 37 |
| 397 | Ho K.-Y. | A prospective study of the clinical features, manometric findings, incidence and prevalence of achalasia in Singapore | Journal of Gastroenterology and Hepatology (Australia) | All | clinical research | 1999 | 37 |
| 398 | Imperiale, TF | A cost-minimization analysis of alternative treatment strategies for achalasia | AMERICAN JOURNAL OF GASTROENTEROLOGY | Treatment | clinical research | 2000 | 36 |
| 399 | Iqbal A. | Technique and follow-up of minimally invasive Heller myotomy for achalasia | Surgical Endoscopy and Other Interventional Techniques | Treatment | clinical research | 2006 | 36 |
| 400 | Shoenut J.P. | Reflux in untreated achalasia patients | Journal of Clinical Gastroenterology | Other | clinical research | 1995 | 36 |
| 401 | Wang L. | Recurrent Achalasia treated with heller myotomy: A review of the literature | World Journal of Gastroenterology | Treatment | review | 2008 | 36 |
| 402 | Meshkinpour H. | Quality of life among patients treated for achalasia | Digestive Diseases and Sciences | Other | clinical research | 1996 | 36 |
| 403 | Borges A.A. | Pneumatic dilation versus laparoscopic Heller myotomy for the treatment of achalasia: Variables related to a good response | Diseases of the Esophagus | Treatment | clinical research | 2014 | 36 |
| 404 | Fraiji Jr. E. | Laparoscopic management of symptomatic achalasia associated with epiphrenic diverticulum | Surgical Endoscopy and Other Interventional Techniques | Treatment | clinical research | 2003 | 36 |
| 405 | Katilius M. | Heller myotomy for achalasia: quality of life comparison of laparoscopic and open approaches. | JSLS : Journal of the Society of Laparoendoscopic Surgeons / Society of Laparoendoscopic Surgeons | Treatment | clinical research | 2001 | 36 |
| 406 | Kahrilas P.J. | Expert consensus document: Advances in the management of oesophageal motility disorders in the era of high-resolution manometry: A focus on Achalasia syndromes | Nature Reviews Gastroenterology and Hepatology | Treatment | clinical research | 2017 | 36 |
| 407 | Shiwaku H. | A prospective analysis of GERD after POEM on anterior myotomy | Surgical Endoscopy | Treatment | clinical research | 2016 | 36 |
| 408 | Bloomston M. | Videoscopic heller myotomy as first-line therapy for severe achalasia | American Surgeon | Treatment | clinical research | 2001 | 35 |
| 409 | Rai R.R. | Rigiflex pneumatic dilation of achalasia without fluoroscopy: A novel office procedure | Gastrointestinal Endoscopy | Treatment | clinical research | 2005 | 35 |
| 410 | Li C. | Peroral endoscopic myotomy for treatment of achalasia in children and adolescents | Journal of Pediatric Surgery | Treatment | clinical research | 2015 | 35 |
| 411 | Graham A.J. | Laparoscopic esophageal myotomy and anterior partial fundoplication for the treatment of achalasia | Annals of Thoracic Surgery | Treatment | clinical research | 1997 | 35 |
| 412 | Kahrilas P.J. | Evaluation of the esophagogastric junction using high resolution manometry and esophageal pressure topography | Neurogastroenterology and Motility | Diagosis | clinical research | 2012 | 35 |
| 413 | Grimes K.L. | Double-scope per oral endoscopic myotomy (POEM): a prospective randomized controlled trial | Surgical Endoscopy | Treatment | clinical research | 2016 | 35 |
| 414 | Khatami S.S. | Does diffuse esophageal spasm progress to achalasia? A prospective cohort study | Digestive Diseases and Sciences | Other | clinical research | 2005 | 35 |
| 415 | Muraji T. | Congenital cricopharyngeal achalasia: Diagnosis and surgical management. | Journal of pediatric surgery | All | clinical research | 2002 | 35 |
| 416 | Mearin F. | Complete lower esophageal sphincter relaxation observed in some achalasia patients is functionally inadequate | American Journal of Physiology - Gastrointestinal and Liver Physiology | Other | clinical research | 2000 | 35 |
| 417 | Gutschow C.A. | Botox, dilation, or myotomy? Clinical outcome of interventional and surgical therapies for achalasia | Langenbeck's Archives of Surgery | Treatment | clinical research | 2010 | 35 |
| 418 | Cho Y.K. | Assessing bolus retention in achalasia using high-resolution manometry with impedance: A comparator study with timed barium esophagram | American Journal of Gastroenterology | Treatment | clinical research | 2014 | 35 |
| 419 | Paladini F. | Age-dependent association of idiopathic achalasia with vasoactive intestinal peptide receptor 1 gene | Neurogastroenterology and Motility | Etiology | basic science research | 2009 | 35 |
| 420 | Furuzawa-Carballeda J. | Achalasia - An Autoimmune Inflammatory Disease: A Cross-Sectional Study | Journal of Immunology Research | Etiology | basic science research | 2015 | 35 |
| 421 | Gockel, Ines | Achalasia-a Disease of Unknown Cause That Is Often Diagnosed Too Late | DEUTSCHES ARZTEBLATT INTERNATIONAL | All | review | 2012 | 34 |
| 422 | Li Q.-L. | Perspective on peroral endoscopic myotomy for achalasia: Zhongshan experience | Gut and Liver | Treatment | clinical research | 2015 | 34 |
| 423 | Lv L. | Peroral endoscopic full-thickness myotomy for the treatment of sigmoid-type Achalasia: Outcomes with a minimum follow-up of 12 months | European Journal of Gastroenterology and Hepatology | Treatment | clinical research | 2016 | 34 |
| 424 | Hirano I. | Pathophysiology of achalasia. | Current gastroenterology reports | Other | basic science research | 1999 | 34 |
| 425 | Li Q.-L. | Outcomes of per-oral endoscopic myotomy for treatment of esophageal achalasia with a median follow-up of 49 months | Gastrointestinal Endoscopy | Treatment | clinical research | 2018 | 34 |
| 426 | Kjellin A.P. | Laparoscopic myotomy without fundoplication in patients with achalasia | European Journal of Surgery | Treatment | clinical research | 1999 | 34 |
| 427 | Alves A. | Laparoscopic Heller's cardiomyotomy in achalasia: Is intraoperative endoscopy useful, and why? | Surgical Endoscopy | Treatment | clinical research | 1999 | 34 |
| 428 | Omura N. | Laparoscopic Heller myotomy and Dor fundoplication for the treatment of achalasia: Assessment in relation to morphologic type | Surgical Endoscopy and Other Interventional Techniques | Treatment | clinical research | 2006 | 34 |
| 429 | Carlson D.A | High-Resolution Manometry and Esophageal Pressure Topography. Filling the Gaps of Convention Manometry | Gastroenterology Clinics of North America | Diagnosis | review | 2013 | 34 |
| 430 | Ip K.S. | Botulinum toxin for achalasia in children | Journal of Gastroenterology and Hepatology (Australia) | Treatment | clinical research | 2000 | 34 |
| 431 | Schneider A.M. | A Matched Comparison of Per Oral Endoscopic Myotomy to Laparoscopic Heller Myotomy in the Treatment of Achalasia | Journal of Gastrointestinal Surgery | Treatment | clinical research | 2016 | 34 |
| 432 | Piotrowska, AP | Distribution of interstitial cells of Cajal in the internal anal sphincter of patients with internal anal sphincter achalasia and Hirschsprung disease | ARCHIVES OF PATHOLOGY & LABORATORY MEDICINE | Other | basic science research | 2003 | 33 |
| 433 | Cai M.-Y. | Thoracic CT after peroral endoscopic myotomy for the treatment of achalasia | Gastrointestinal Endoscopy | Treatment | clinical research | 2014 | 33 |
| 434 | Molena D. | Surgical Management of End-Stage Achalasia | Seminars in Thoracic and Cardiovascular Surgery | Treatment | review | 2012 | 33 |
| 435 | Shiino Y. | Surgery for Achalasia: 1998 | Journal of Gastrointestinal Surgery | Treatment | review | 1999 | 33 |
| 436 | Ferguson M.K. | Results of myotomy and partial fundoplication after pneumatic dilation for achalasia | Annals of Thoracic Surgery | Treatment | clinical research | 1996 | 33 |
| 437 | Johnston B.T. | Repetitive proximal esophageal contractions: A new manometric finding and a possible further link between Parkinson's disease and achalasia | Dysphagia | Other | clinical research | 2001 | 33 |
| 438 | Minami H. | Per-oral endoscopic myotomy: Emerging indications and evolving techniques | Digestive Endoscopy | Treatment | review | 2015 | 33 |
| 439 | Stavropoulos S.N. | Per-oral endoscopic myotomy white paper summary | Surgical Endoscopy | Treatment | Guideline and consensus | 2014 | 33 |
| 440 | Mearin F. | Impaired gastric relaxation in patients with achalasia | Gut | Other | clinical research | 1995 | 33 |
| 441 | Miller L.S. | High-resolution endoluminal sonography in achalasia | Gastrointestinal Endoscopy | Diagnosis | clinical research | 1995 | 33 |
| 442 | Kostic S. | Health economic evaluation of therapeutic strategies in patients with idiopathic achalasia: Results of a randomized trial comparing pneumatic dilatation with laparoscopic cardiomyotomy | Surgical Endoscopy and Other Interventional Techniques | Other | clinical research | 2007 | 33 |
| 443 | Finley C.J. | Factors Associated With Postoperative Symptoms After Laparoscopic Heller Myotomy | Annals of Thoracic Surgery | Treatment | clinical research | 2010 | 33 |
| 444 | Tan Y. | Efficacy of anterior versus posterior per-oral endoscopic myotomy for treating achalasia: a randomized, prospective study | Gastrointestinal Endoscopy | Treatment | clinical research | 2018 | 33 |
| 445 | Villanacci V. | An immunohistochemical study of the myenteric plexus in idiopathic achalasia | Journal of Clinical Gastroenterology | Etiology | Basic science research | 2010 | 33 |
| 446 | Nabi, Zaheer | Per-oral endoscopic myotomy for achalasia cardia: outcomes in over 400 consecutive patients | ENDOSCOPY INTERNATIONAL OPEN | Treatment | clinical research | 2017 | 33 |
| 447 | Ramacciato G. | The laparoscopic approach with antireflux surgery is superior to the thoracoscopic approach for the treatment of esophageal achalasia: Experience of a single surgical unit | Surgical Endoscopy and Other Interventional Techniques | Treatment | clinical research | 2002 | 32 |
| 448 | Khan A.A. | Sixteen years follow up of achalasia: A prospective study of graded dilatation using Rigiflex ballon | Diseases of the Esophagus | Treatment | clinical research | 2005 | 32 |
| 449 | Tanaka Y. | Predictors of a better outcome of pneumatic dilatation in patients with primary achalasia | Journal of Gastroenterology | Treatment | clinical research | 2010 | 32 |
| 450 | Yang S. | Pneumomediastinum and pneumoperitoneum on computed tomography after peroral endoscopic myotomy (POEM): Postoperative changes or complications? | Acta Radiologica | Treatment | clinical research | 2015 | 32 |
| 451 | Zhang W. | Peroral Endoscopic Myotomy for Type III Achalasia of Chicago Classification: Outcomes with a Minimum Follow-Up of 24 Months | Journal of Gastrointestinal Surgery | Treatment | clinical research | 2017 | 32 |
| 452 | Kumagai K. | Per-oral endoscopic myotomy for achalasia. Are results comparable to laparoscopic Heller myotomy? | Scandinavian Journal of Gastroenterology | Treatment | clinical research | 2015 | 32 |
| 453 | Bredenoord A.J | Peroral endoscopic myotomy for achalasia | Neurogastroenterology and Motility | Treatment | review | 2014 | 32 |
| 454 | Gockel I. | Long-term Results of Conventional Myotomy in Patients With Achalasia: A Prospective 20-Year Analysis | Journal of Gastrointestinal Surgery | Treatment | clinical research | 2006 | 32 |
| 455 | Lin Z. | High-resolution impedance manometry measurement of bolus flow time in achalasia and its correlation with dysphagia | Neurogastroenterology and Motility | Diagnosis | clinical research | 2015 | 32 |
| 456 | Werner Y.B. | Early adverse events of per-oral endoscopic myotomy | Gastrointestinal Endoscopy | Treatment | clinical research | 2017 | 32 |
| 457 | Moonen A. | Current diagnosis and management of achalasia | Journal of Clinical Gastroenterology | All | review | 2014 | 32 |
| 458 | van Hoeij, F. B. | Clinical application of esophageal high-resolution manometry in the diagnosis of esophageal motility disorders | Journal of Neurogastroenterology and Motility | Diagnosis | clinical research | 2016 | 32 |
| 459 | Alonso Aguirre P. | Achalasia. The usefulness of manometry for evaluation of treatment | Digestive Diseases and Sciences | Treatment | clinical research | 1999 | 32 |
| 460 | Storr M., Born P. | Treatment of achalasia: The short-term response to botulinum toxin injection seems to be independent of any kind of pretreatment | BMC Gastroenterology | Treatment | clinical research | 2002 | 32 |
| 461 | Brooks A. | The surgical management of cricopharyngeal achalasia in children | International Journal of Pediatric Otorhinolaryngology | Treatment | clinical research | 2000 | 31 |
| 462 | Duffy P.E. | The laparoscopic reoperation of failed Heller myotomy | Surgical Endoscopy and Other Interventional Techniques | Treatment | clinical research | 2003 | 31 |
| 463 | Karanicolas P.J. | The cost of laparoscopic myotomy versus pneumatic dilatation for esophageal achalasia | Surgical Endoscopy and Other Interventional Techniques | Treatment | clinical research | 2007 | 31 |
| 464 | Di Nardo G. | Pneumatic balloon dilation in pediatric achalasia: Efficacy and factors predicting outcome at a single tertiary pediatric gastroenterology center | Gastrointestinal Endoscopy | Treatment | clinical research | 2012 | 31 |
| 465 | Mehra M. | Laparoscopic and thoracoscopic esophagomyotomy for children with achalasia | Journal of Pediatric Gastroenterology and Nutrition | Treatment | clinical research | 2001 | 31 |
| 466 | Robson K. | GERD progressing to diffuse esophageal spasm and then to achalasia | Digestive Diseases and Sciences | Other | clinical research | 2000 | 31 |
| 467 | Kornblum C. | Cricopharyngeal achalasia is a common cause of dysphagia in patients with mtDNA deletions | Neurology | Etiology | basic science research | 2001 | 31 |
| 468 | Roberts K.E. | Controversies in the treatment of gastroesophageal reflux and achalasia | World Journal of Gastroenterology | Treatment | review | 2006 | 31 |
| 469 | Richter J.E. | Comparison and cost analysis of different treatment strategies in achalasia | Gastrointestinal Endoscopy Clinics of North America | Treatment | review | 2001 | 31 |
| 470 | Savarino E. | Achalasia with dense eosinophilic infiltrate responds to steroid therapy | Clinical Gastroenterology and Hepatology | Treatment | clinical research | 2011 | 31 |
| 471 | Peters, JH | An antireflux procedure is critical to the long-term outcome of esophageal myotomy for achalasia | JOURNAL OF GASTROINTESTINAL SURGERY | Treatment | clinical research | 2001 | 31 |
| 472 | González M. | Oesophageal tone in patients with achalasia | Gut | Other | clinical research | 1997 | 30 |
| 473 | Delgado F. | Laparoscopic treatment of esophageal achalasia | Surgical Laparoscopy, Endoscopy and Percutaneous Techniques | Treatment | clinical research | 1996 | 30 |
| 474 | Patti M.G. | Laparoscopic heller myotomy and dor fundoplication for esophageal achalasia. How i do it | Journal of Gastrointestinal Surgery | Treatment | clinical research | 2008 | 30 |
| 475 | Mitchell P.C. | Laparoscopic cardiomyotomy with a Dor patch for achalasia | Canadian Journal of Surgery | Treatment | clinical research | 1995 | 30 |
| 476 | Bessell J.R. | Laparoscopic cardiomyotomy for achalasia: Long-term outcomes | ANZ Journal of Surgery | Treatment | clinical research | 2006 | 30 |
| 477 | DaǧlI Ü. | Factors predicting outcome of balloon dilatation in achalasia | Digestive Diseases and Sciences | Treatment | clinical research | 2009 | 30 |
| 478 | Dantas R.O. | Esophageal motility of patients with Chagas' disease and idiopathic achalasia | Digestive Diseases and Sciences | Other | clinical research | 2001 | 30 |
| 479 | Yaghoobi M. | Correlation between clinical severity score and the lower esophageal sphincter relaxation pressure in idiopathic achalasia | American Journal of Gastroenterology | Other | clinical research | 2003 | 30 |
| 480 | Hamza A.F. | Cardiac achalasia in children. Dilatation or surgery? | European Journal of Pediatric Surgery | Treatment | clinical research | 1999 | 30 |
| 481 | Watanabe Y. | Attenuated nitrergic inhibitory neurotransmission to interstitial cells of Cajal in the lower esophageal sphincter with esophageal achalasia in children | Pediatrics International | Etiology | basic science research | 2002 | 30 |
| 482 | Tyberg A. | A multicenter international registry of redo per-oral endoscopic myotomy (POEM) after failed POEM | Gastrointestinal Endoscopy | treatment | clinical research | 2017 | 30 |

**Table S1** The eligible articles in achalasia research
